# CGRP sensory neurons promote tissue healing via neutrophils and macrophages

**DOI:** 10.1038/s41586-024-07237-y

**Published:** 2024-03-27

**Authors:** Yen-Zhen Lu, Bhavana Nayer, Shailendra Kumar Singh, Yasmin K. Alshoubaki, Elle Yuan, Anthony J. Park, Kenta Maruyama, Shizuo Akira, Mikaël M. Martino

**Affiliations:** 1 European Molecular Biology Laboratory Australia, Australian Regenerative Medicine Institute, Monash University, Melbourne, Victoria Australia; 2 Laboratory of Host Defense, World Premier International Research Center, Immunology Frontier Research Center, Osaka University, Osaka, Japan; 3 Department of Pharmacology, School of Medicine, Aichi Medical University, Aichi, Japan; 4 Victorian Heart Institute, Monash University, Melbourne, Victoria Australia

**Keywords:** Regenerative medicine, Neuroimmunology

## Abstract

The immune system has a critical role in orchestrating tissue healing. As a result, regenerative strategies that control immune components have proved effective^1,2^. This is particularly relevant when immune dysregulation that results from conditions such as diabetes or advanced age impairs tissue healing following injury^2,3^. Nociceptive sensory neurons have a crucial role as immunoregulators and exert both protective and harmful effects depending on the context^4–12^. However, how neuro–immune interactions affect tissue repair and regeneration following acute injury is unclear. Here we show that ablation of the Na_V_1.8 nociceptor impairs skin wound repair and muscle regeneration after acute tissue injury. Nociceptor endings grow into injured skin and muscle tissues and signal to immune cells through the neuropeptide calcitonin gene-related peptide (CGRP) during the healing process. CGRP acts via receptor activity-modifying protein 1 (RAMP1) on neutrophils, monocytes and macrophages to inhibit recruitment, accelerate death, enhance efferocytosis and polarize macrophages towards a pro-repair phenotype. The effects of CGRP on neutrophils and macrophages are mediated via thrombospondin-1 release and its subsequent autocrine and/or paracrine effects. In mice without nociceptors and diabetic mice with peripheral neuropathies, delivery of an engineered version of CGRP accelerated wound healing and promoted muscle regeneration. Harnessing neuro–immune interactions has potential to treat non-healing tissues in which dysregulated neuro–immune interactions impair tissue healing.

## Abstract (web summary)

Experiments in mouse models show that Na_V_1.8^+^ nociceptors innervate sites of injury and provide wound repair signals to immune cells by releasing calcitonin gene-related peptide (CGRP).

## Main

The design of successful regenerative medicine treatments requires leveraging the key factors that have pivotal roles in tissue healing. Modulating the immune system to promote tissue healing has demonstrated to be remarkably effective, since immune components coordinate a complex series of events that are critical for proper healing^1,2^. Unsurprisingly, the immune dysregulation that occurs as a consequence of ageing or conditions such as diabetes has a substantial negative effect on tissue healing and is often characterized by persistent inflammation at the injured site^2,3^. Another potentially important factor in tissue repair and regeneration is the nervous system. For instance, studies using neurotomy models have shown that peripheral nerves have an essential role in some animal species that are capable of regenerating tissues^13–16^. Other studies using nerve depletion in mouse, which are more specific to sensory neurons, have shown that nociceptive neurons and non-peptidergic C-fibres that express Gα_i_-interacting protein promote adipose tissue regeneration^17^ and skin repair after UV-induced damage^18^, respectively. Nociceptive sensory neurons (also referred to as nociceptors) are specialized primary sensory neurons that have nerve endings in tissues such as skin, muscles and joints. They detect and respond to noxious stimuli including temperature, chemicals and inflammatory mediators^19^. Nociceptors have been shown to be critical immunoregulators with both protective and harmful effects^4,20,21^. For example, nociceptors either diminish or exacerbate inflammation, enhance resistance to pathogens, or impede their clearance^5–12^. Therefore, given the immunomodulatory properties of nociceptors and the central role of the immune system in tissue repair and regeneration, we investigated the importance of peptidergic sensory neurons in tissue healing following acute injury, and examined whether neuro–immune interactions could be harnessed to promote tissue healing. Specifically, we used skin and muscle as relevant tissue models that are known to be innervated by nociceptors and in which repair and regeneration outcomes are considerably modulated by the immune system^1–3,22,23^.

## Tissue healing in the absence of sensory neurons

**Fig. 1:**
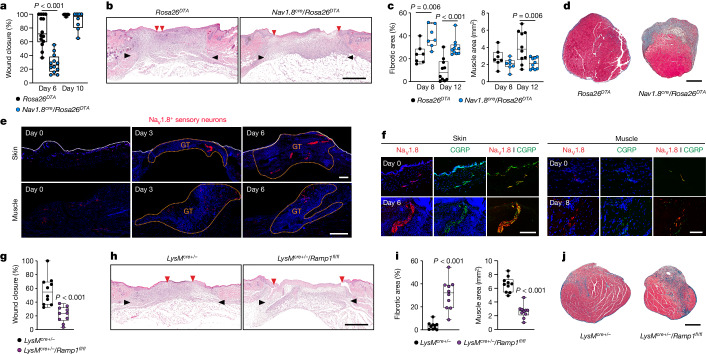
Na_V_1.8^+^ nociceptors that express CGRP mediate tissue healing via myeloid cells. **a**,**b**, Full-thickness skin wounds were created in denervated (*Nav1.8*^*cre*^/*Rosa26*^*DTA*^) mice and littermate controls (*Rosa26*^*DTA*^). **a**, Wound closure evaluated by histomorphometric analysis on day 6 and day 10 post-injury (*Rosa26*^*DTA*^ day 6, *n* = 14; *Nav1.8*^*cre*^/*Rosa26*^*DTA*^ day 6, *n* = 12; day 10, *n* = 8). **b**, Representative histology at day 6 post-injury. Black arrows indicate wound edges and red arrows indicate tips of epithelium tongue. Scale bar, 1 mm. **c**,**d**, Volumetric muscle loss was evaluated on quadriceps of *Nav1.8*^*cre*^/*Rosa26*^*DTA*^ and *Rosa26*^*DTA*^ littermate controls. **c**, Extent of muscle regeneration was evaluated by histomorphometric analysis on day 8 and day 12 post-injury (day 8, *n* = 7; day 12, *n* = 10). **d**, Representative histology at day 12 post-injury (fibrotic tissue is stained blue; muscle tissue is stained red). Scale bar, 500 μm. **e**, Distribution of Na_V_1.8^+^ sensory neurons (red) in skin and muscle of *Nav1.8*^*cre*^/*Rosa26*^*tdT*^ mice before and after injury. White lines indicate keratinocyte layers and nuclei are in blue. GT, granulation tissue area. Scale bars, 500 μm. The experiment was repeated independently four times. **f**, Expression of CGRP in skin and muscle before and after injury detected by immunohistochemistry. Scale bars: 500 μm (skin), 100 μm (muscle). The experiment was repeated independently four times. **g**–**j**, *Ramp1* deletion in myeloid cells using *LysM*^*cre*^^*+/−*^/*Ramp1*^*fl/fl*^ mice. *LysM*^*cre*^^*+/−*^ mice were used as controls. **g**, Wound closure was quantified on day 6 post-injury (*n* = 10). **h**, Representative skin histology. **i**, Muscle regeneration was quantified on day 12 post-injury (*n* = 10). **j**, Representative muscle histology. Boxes show median (centre line) and interquartile range (edges), whiskers show the range of values and dots represent individual data points. **a**,**c**, Two-way ANOVA with Bonferroni post hoc test for pairwise comparisons. **g**,**i**, Two-tailed Student’s *t-*test. *P* values are indicated. Source Data

**Extended Data Fig. 1:**
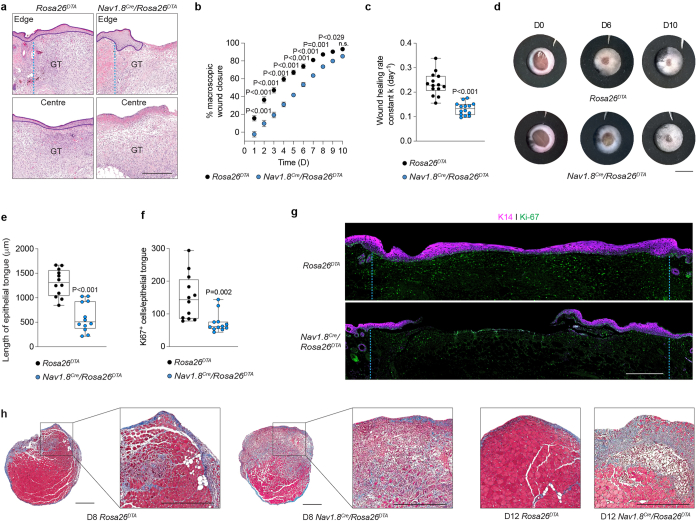
The lack of Na_V_1.8^+^ sensory neurons impairs tissue healing. **a–g**, Full-thickness skin wounds were created in *Nav1.8*^*cre*^*/Rosa26*^*DTA*^ and littermate control *Rosa26*^*DTA*^ mice. Wound closure was evaluated by histomorphometric analysis of tissue sections at D6 post-injury. Representative histology at the edge and centre of the wounds (**a**). Blue lines indicate wound edges. Purple lines indicate the epithelial tongue. GT indicates granulation tissue. Scale bar = 500 μm. Wound closure was evaluated by macroscopic evaluation (**b**) to calculate the wound healing rate constant κ (**c**) (*n* = 14). Representative macroscopic images of the wounds (**d**). Scale bar = 5 mm. Measurement of the length of the migrating epithelial tongue (**e**) (*n* = 12). Quantification of the number of proliferating keratinocytes (Ki-67 positive) in tissue sections (**f**) (*n* = 12). Representative image of immunostaining for keratin 14 (K14, purple) detecting the epithelial tongue and Ki-67 positive cells (green) (**g**). Blue lines indicate wound edges. Scale bar = 500 μm. In **b**, data are plotted in kinetic line plots showing mean ± SEM. In **c,e,f**, data are plotted in box plots showing median (centre line) and IQR (bounds). Whiskers show min. to max. range. Dots represent individual wounds. Two-way ANOVA with Bonferroni *post hoc* test for pair-wise comparisons in **b**. Two-tailed Student’s *t-*test in **c**,**e,f**. *P* values are indicated; n.s., non-significant. **h**, Volumetric muscle loss was performed on the quadriceps of *Nav1.8*^*Cre*^/*Rosa26*^*DTA*^ and *Rosa26*^*DTA*^ littermate control mice. Representative histology is shown (fibrotic tissue is stained blue; muscle tissue is stained red). Scale bars = 500 μm. Repeated independently 7 times for D8 and 10 times for D12. Source Data

To determine the importance of sensory neurons during tissue healing after acute injury, we used the *Nav1.8*^*cre*^*/Rosa26*^*DTA*^ mouse. In this mouse, Na_V_1.8^+^ dorsal root ganglion (DRG) neurons, which mainly represent nociceptors involved in mechanical, cold and inflammatory pain, are ablated by the expression of diphtheria toxin fragment A^5,7,8,10,12,24^ (DTA). *Rosa26*^*DTA*^ littermates with intact Na_V_1.8-expressing sensory neurons were used as controls. As acute injury models, we selected full-thickness wounds in the dorsal skin^25,26^ and volumetric muscle loss injuries in quadriceps^23^. The absence of Na_V_1.8-expressing sensory neurons resulted in a significant delay in skin wound closure, which was evident through decreased epithelial migration, a reduced number of proliferative keratinocytes and wounds remaining largely open after six days (Fig. 1a,b and Extended Data Fig. 1a–g). Similarly, muscle regeneration was impaired in *Nav1.8*^*cre*^*/Rosa26*^*DTA*^ mice, which were characterized by increased fibrotic tissue and reduced muscle tissue formation (Fig. 1c,d and Extended Data Fig. 1h).

**Extended Data Fig. 2:**
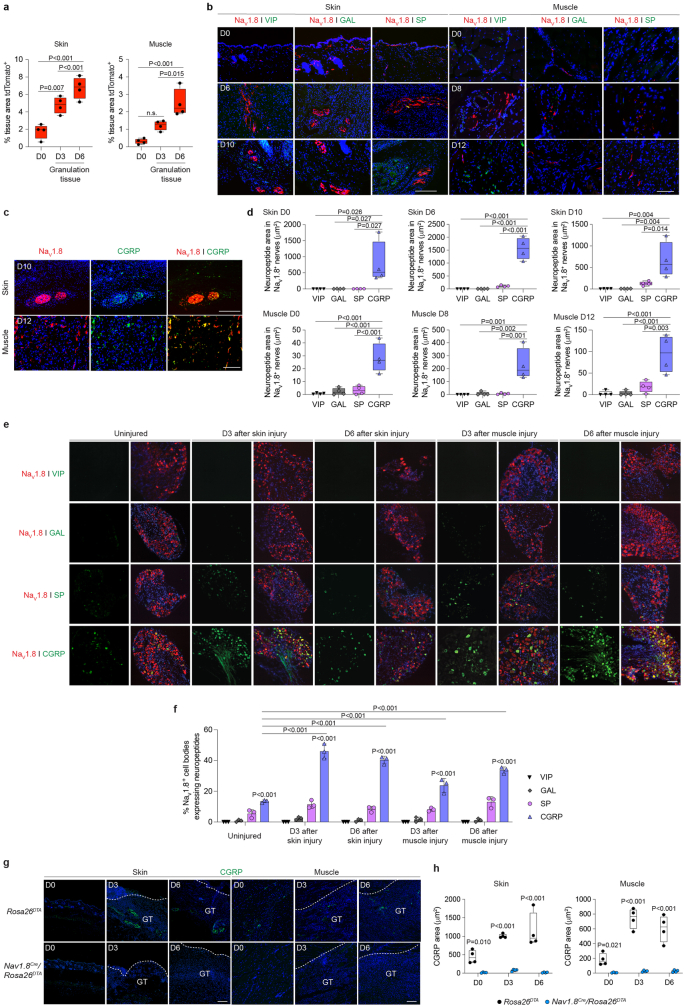
Na_V_1.8^+^ sensory neurons mainly express CGRP during skin and muscle healing. **a**, Quantification of Na_V_1.8^+^ sensory neurons in skin and muscle before and after injury. Results are expressed as the percentage of tissue area positive for tdTomato in healthy tissue at D0 (uninjured) and in the granulation tissue at D3 and D6 post-injury (*n* = 4). **b–d**, Expression of neuropeptides in skin and muscle were detected by immunohistochemistry (neuropeptides, green; Na_V_1.8, red; nuclei, blue). Scale bars = 500 μm in skin and 100 μm in muscle. Quantification of neuropeptide signal in Na_V_1.8^+^ sensory neurons (**d**) (*n* = 4). **e**, Neuropeptide expression in DRGs before and on D3 and D6 after skin and muscle injury (neuropeptides, green; Na_V_1.8, red; nuclei, blue). Scale bar = 100 μm. Percentage of Na_V_1.8^+^ cell bodies expressing a neuropeptide type (**f**) (*n* = 3). **g**, CGRP localisation in skin and muscle before and after tissue injury in *Nav1.8*^*Cre*^/*Rosa26*^*DTA*^ and *Rosa26*^DTA^ littermate control mice. White lines indicate the separation between the granulation tissue (GT) and the top of the wound in skin and the separation between the granulation tissue and the muscle tissue in muscle. Scale bars = 100 μm. Quantification of CGRP signal (**h**) (*n* = 4). In **a,d,h**, data are plotted in box plots showing median (centre line) and IQR (bounds). Whiskers show min. to max. range. Dots represent independent injuries. In **f**, data are plotted as bar graph showing mean ± SD. Dots represent individual DRGs. One-way ANOVA with Tukey *post hoc* test for pair-wise comparisons in **a,d**. Two-way ANOVA with Bonferroni *post hoc* test for pair-wise comparisons in **f,h**. *P* values are indicated. Source Data

Nociceptor terminals release neuropeptides in response to danger signals that include inflammatory cytokines commonly present in tissues after acute injury^27,28^. Thus, we investigated the distribution of Na_V_1.8^+^ sensory neurons during skin and muscle healing following acute injury. To visualize Na_V_1.8^+^ sensory neuron distribution and the neuropeptides that they express, we used *Nav1.8*^*cre*^/*Rosa26*^*tdT*^ mice where tdTomato fluorescent protein expression is restricted to Na_V_1.8^+^ sensory neurons. Tissue sections of *Nav1.8*^*cre*^/*Rosa26*^*tdT*^ uninjured skin and muscle showed Na_V_1.8^+^ sensory neuron distribution across the epidermis and dermis in skin and nearby connective tissues in muscle (Fig. 1e). Following skin and muscle injury, we observed that Na_V_1.8^+^ sensory neuron endings growing in clusters into the granulation tissue, thus establishing innervation within the injured area during the healing process (Fig. 1e and Extended Data Fig. 2a). Na_V_1.8^+^ neurons exhibited expression of calcitonin gene-related peptide (CGRP) and substance P, and no detectable expression of vasoactive intestinal peptide (VIP) or galanin (GAL) (Fig. 1f and Extended Data Fig. 2b–d). Similarly, in the DRG following both skin and muscle injuries, these neurons showed expression of CGRP and substance P in cell bodies (Extended Data Fig. 2e,f). Additionally, the absence of CGRP signal in *Nav1.8*^*cre*^*/Rosa26*^*DTA*^ mice pre- and post-tissue injury underscores the role of Na_V_1.8^+^ nociceptors as primary sources of CGRP during skin and muscle healing and suggests that nociceptor-derived CGRP has a pivotal role in these processes (Extended Data Fig. 2g,h).

## CGRP effect on injury immune cells

**Extended Data Fig. 3:**
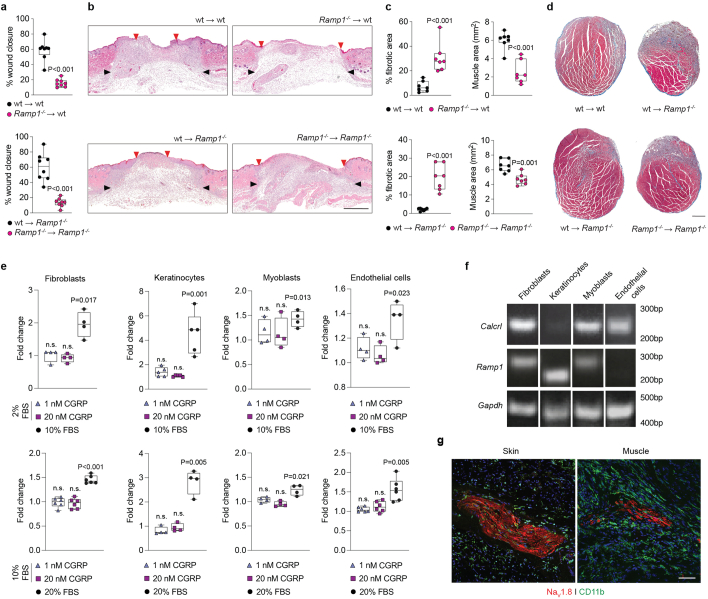
CGRP signalling in immune cells mediates skin and muscle healing. **a–d**, Wildtype (wt) or *Ramp1*^*–/–*^ mice were γ-irradiated and received a bone marrow transplant from wt or *Ramp1*^–/–^ donor mice. Full-thickness skin wounds or quadriceps volumetric muscle loss were performed. Wound closure was evaluated by histomorphometric analysis of tissue sections at D6 post-injury (**a**) (*n* = 8). Representative histology (**b**). Black arrows indicate wound edges. Red arrows indicate tips of epithelium tongue. Scale bar = 1 mm. The extent of muscle regeneration (represented by the percentage of fibrotic tissue and muscle area) was evaluated by histomorphometric analysis of tissue sections at D12 post-injury (**c**) (*n* = 7). Representative histology D12 post-injury (fibrotic tissue is stained blue; muscle tissue is stained red) (**d**). Scale bar = 500 μm. **e**, Cell proliferation in response to CGRP treatment (2% FBS: *n* = 5 for keratinocytes, *n* = 4 for all other cell types; 10% FBS: *n* = 6 for fibroblasts and endothelial cells, *n* = 4 for keratinocytes and myoblasts). Foetal bovine serum (FBS, 10%–20%) was used as a positive control and results are expressed as fold change over saline control (0 nM CGRP with 2% or 10% FBS). For gel source data, see Supplementary Fig. 1. **f**, *Calcrl* and *Ramp1* expression in fibroblasts, keratinocytes, myoblasts, and endothelial cells, detected by RT-PCR. *Gapdh* was used as the housekeeping gene. Repeated independently 3 times. **g**, Representative images showing the close proximity of CD11b^+^ cells with Na_V_1.8^+^ sensory neurons in granulation tissue 6D after skin and muscle injury (CD11b, green; Na_V_1.8, red; nuclei, blue). Scale bar = 50 μm. Repeated independently 4 times. All data are plotted in box plots showing median (centre line) and IQR (bounds). Whiskers show min. to max. range. Dots represent individual injuries or experiments. Two-tailed Student’s *t*-test in **a,c**. Two-tailed one-sample *t*-test over fold increase of 1 in **e**. *P* values are indicated; n.s., non-significant. Source Data

To investigate whether CGRP mediates neuro–immune interactions that drive tissue healing, we generated mice in which immune cells or non-immune cells were unable to respond to CGRP. Bone marrow cells deficient for RAMP1^29^, a co-receptor that is essential for CGRP signalling, were transplanted into γ-irradiated wild-type or *Ramp1*^*−/−*^ mice. Wild-type mice that received wild-type bone marrow cells or *Ramp1*^*−/−*^ mice that received *Ramp1*^*−/−*^ bone marrow cells were used as controls. Skin and muscle healing were severely impaired in wild-type mice reconstituted with *Ramp1*^*−/−*^ cells compared with those with wild-type cells. Similarly, transfer of *Ramp1*^*−/−*^ cells into *Ramp1*^*−/−*^ mice impaired healing, whereas wild-type cells rescued skin repair and muscle regeneration (Extended Data Fig. 3a–d). Considering that myeloid cells such as neutrophils and monocytes/macrophages constitute the majority of immune cells in injured tissues undergoing repair or regeneration and can represent up to 50% of the total wound cells^1,2,23,26^, we hypothesized that CGRP promotes tissue healing by modulating myeloid cells. To investigate this, we produced mice that lacked CGRP signalling in myeloid cells by crossing *LysM*^*cre*^ (*LyzM* is also known as *Lyz2*) mice with *Ramp1* floxed (*Ramp1*^*fl/fl*^) mice^30^. The *LysM*^*cre+/*^^−^/*Ramp1*^*fl/fl*^ mice displayed a significant reduction in skin wound closure and muscle regeneration, compared with *LysM*^*cre*^^+/−^ control mice (Fig. 1g–j). The extent of healing impairment, characterized by a significant delay in skin wound closure and compromised muscle regeneration with high level of fibrotic area, was indeed very similar to that observed in *Nav1.8*^*cre*^/*Rosa26*^*tdT*^ mice. Together, these results suggested that CGRP from Na_V_1.8^+^ sensory neurons promotes tissue healing via myeloid cells. In addition, CGRP treatment in vitro had no significant effect on the proliferation of key cell types involved in skin and muscle healing, including fibroblasts, keratinocytes, myoblasts and endothelial cells (Extended Data Fig. 3e). Further supporting this observation, we found that keratinocytes and myoblasts have low expression levels of either *Ramp1* or calcitonin receptor-like receptor (*Calcrl*), which together form the CGRP receptor complex (Extended Data Fig. 3f). Finally, Na_V_1.8^+^ nociceptors in granulation tissue were found to be surrounded by CD11b^+^ myeloid cells, which consist mainly of neutrophils and monocytes/macrophages in the context of tissue healing after acute injury (Extended Data Fig. 3g).

**Fig. 2:**
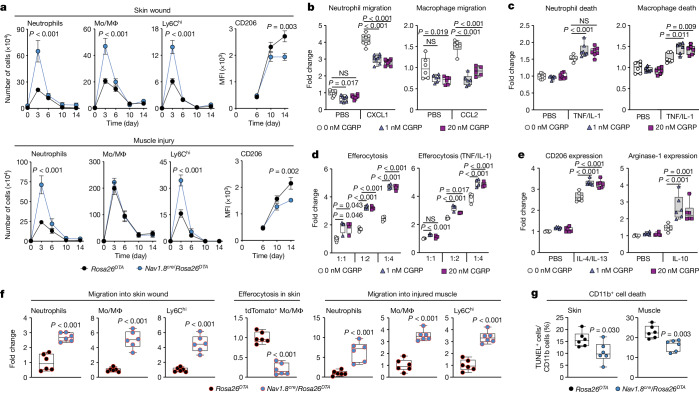
CGRP regulates myeloid cell function during tissue healing. **a**, Analysis of neutrophil and monocyte/macrophage (Mo/Mϕ) populations by flow cytometry during tissue healing. Geometric mean of fluorescence intensity (MFI) of CD206 in macrophages was used to assess M2-like polarization. Data are plotted in kinetic line plots showing mean ± s.e.m. Skin: day 0, day 6 and day 10, *n* = 20; day 3, *n* = 22; and day 14, *n* = 12. Muscle: day 0, *n* = 16; day 3, *n* = 20; day 6, day 10, *n* = 18; and day 14, *n* = 10. **b**–**e**, Neutrophils and macrophages were treated with saline (PBS, 0 nM CGRP) or CGRP (1 or 20 nM). Results are expressed as fold change over the PBS (0 nM CGRP) group. **b**, Transwell migration towards CXCL1 or CCL2 with or without CGRP (*n* = 6–8). **c**, Cell death in response to CGRP and TNF plus IL-1 (neutrophils, *n* = 7; macrophages, *n* = 6). **d**, Macrophage efferocytosis of neutrophils after CGRP treatment with or without TNF/IL-1 (*n* = 4). **e**, Macrophage polarization determined via CD206 and arginase-1 protein expression following CGRP and IL-4/IL-13 or IL-10 treatment (*n* = 6). **f**, tdTomato^+^ bone marrow cells were administered systemically on day 2 post-injury. Fold change of tdTomato^+^ neutrophils and monocytes/macrophages in injured tissues on day 3 post-injury was assessed by flow cytometry. For efferocytosis, dead or dying tdTomato^+^ neutrophils were injected in skin wound borders on day 3 post-injury. Fold change of tdTomato^+^ endogenous monocytes/macrophages was assessed by flow cytometry 30 min post-injection (*n* = 6). **g**, Death of CD11b^+^ cells 3 days post-injury, assessed by TUNEL assay on injured tissue sections (*n* = 6). Boxes show median (centre line) and interquartile range (edges), whiskers show the range of values. Dots represent independent experiments. **a**–**e**, Two-way ANOVA with Bonferroni post hoc test for pairwise comparisons. **f**,**g**, Two-tailed Student’s *t-*test. *P* values are indicated. NS, not significant. Source Data

**Extended Data Fig. 4:**
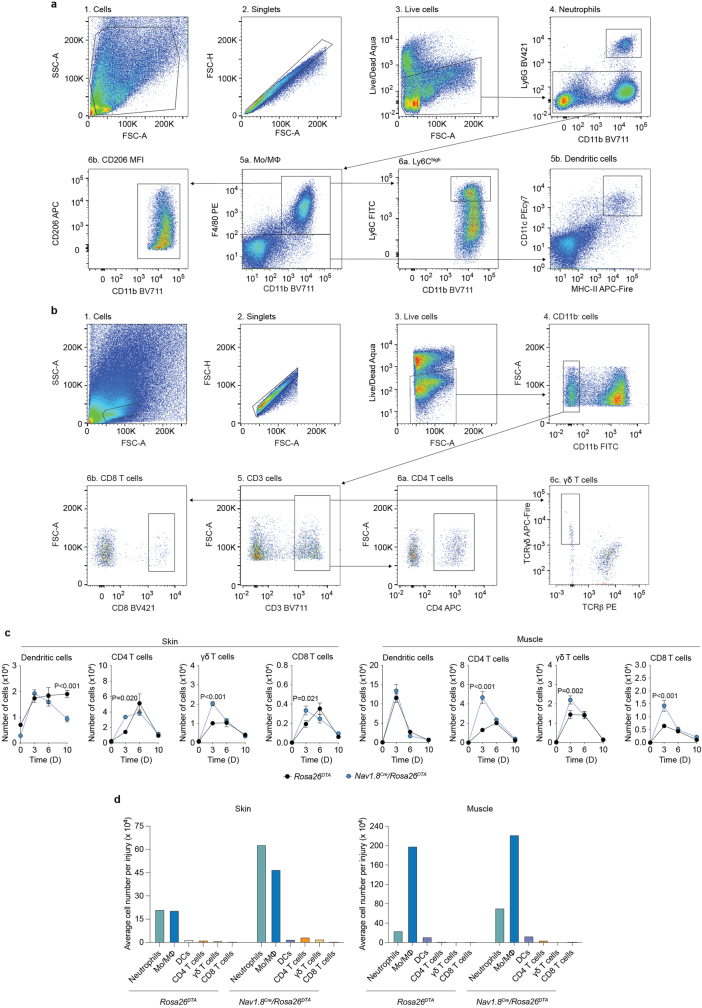
Analyse wound immune cell dynamics in injured skin and muscle. **a**, Gating strategy to analyse myeloid cells in skin and muscle post-injury. Flow cytometry dot plots representing the step by step (1–6) gating strategy to identify neutrophil (CD11b^+^, Ly6G^+^, F4/80^–^) monocytes/macrophages (Mo/Mϕ; CD11b^+^, F4/80^+,^ Ly6G^–^), and dendritic cells (CD11c^+^, MHC-II^+^). MFI is the geometric-mean of fluorescence intensity. **b**, Gating strategy to analyse T cells in skin and muscle post-injury. Flow cytometry dot plots representing the step by step (1–6) gating strategy to identify CD4 T cells (CD3^+^, CD4^+^), cytotoxic T cells (CD3^+^, CD8^+^), and γδ T cells (CD3^+^, TCRβ^–^, TCRγδ^+^). **c**, Numbers of dendritic and T cells in skin and muscle injuries analysis measured by flow cytometry. Data are plotted in kinetic line plots showing mean ± SEM (Skin: *n* = 8 for D0, *n* = 10 for the other time points. Muscle: *n *= 8). Two-way ANOVA with Bonferroni *post hoc* test for pair-wise comparisons. *P* values are indicated; n.s., non-significant. **d**, Bar graph representation of the average number of neutrophils, Mo/Mϕ, dendritic cells, and T cells in injured tissue at D3 post-injury in *Rosa26*^*DTA*^ and *Nav1.8*^*Cre*^/*Rosa26*^*DTA*^ mice. Source Data

To gain insights into the effect of Na_V_1.8^+^ sensory neurons on immune cells during tissue healing, we analysed immune cell dynamics in injured tissues by flow cytometry. We focused on neutrophils, monocytes/macrophages, dendritic cells and T cells, because they constitute the predominant immune populations during tissue healing^1,2,23,26^ (Extended Data Fig. 4a,b). Compared with control mice, *Nav1.8*^*cre*^/*Rosa26*^*DTA*^ mice exhibited an increased number of neutrophils and pro-inflammatory Ly6C^hi^ monocytes/macrophages in skin and muscle 3 days post-injury (Fig. 2a). Similarly, macrophages in *Nav1.8*^*cre*^/*Rosa26*^*DTA*^ mice showed a delayed polarization towards an anti-inflammatory and pro-repair (M2-like) phenotype in both tissues, characterized by a lower expression of CD206, a well-established M2-like macrophage marker, at later stages of the healing process (Fig. 2a). Although the total number of monocytes/macrophages observed 3 days post-injury in the skin was higher in *Nav1.8*^*cre*^/*Rosa26*^*DTA*^ mice compared with controls (Fig. 2a), this phenomenon was not evident in muscle, a difference that may be attributed to either the lower density of Na_V_1.8^+^ nociceptors in injured muscle or variations in the dynamic accumulation of monocytes/macrophages between the two tissues. In both skin and muscle tissues, no major differences were observed in dendritic cells except for a lower number in the skin of *Nav1.8*^*cre*^/*Rosa26*^*DTA*^ at a late time point (day 10 post-injury). However, some variations were noted in the counts of CD4 T cells, γδ T cells and cytotoxic (CD8) T cells 3 days post-injury (Extended Data Fig. 4c). Nevertheless, although these cell populations are recognized for their role in modulating tissue healing, their proportions were considerably lower in both *Rosa26*^*DTA*^ and *Nav1.8*^*cre*^/*Rosa26*^*DTA*^ tissues. They constituted 20–50 times fewer cells compared with neutrophils and monocytes/macrophages in skin and muscle, respectively (Extended Data Fig. 4d). Thus, although CGRP signalling on T cells may affect tissue healing to some extent, the profound impairment of tissue healing upon conditional *Ramp1* knockout in myeloid cells suggests nociceptor-derived CGRP promotes tissue healing after acute injury primarily by influencing myeloid cells (Fig. 1g–j). Overall, the data demonstrated that the absence of nociceptors resulted in an increase in neutrophils and inflammatory monocytes/macrophages. This in turn delayed the transition towards an anti-inflammatory and pro-repair phase^1,2,26^, leading to impaired tissue healing. Of note, these findings align with previous reports showing that nociceptor ablation leads to an increased local accumulation of neutrophils after skin^5,8^ and lung^9^ infection, as well as neutrophils and inflammatory macrophages in colitis^10^ and brain infection^12^.

**Extended Data Fig. 5:**
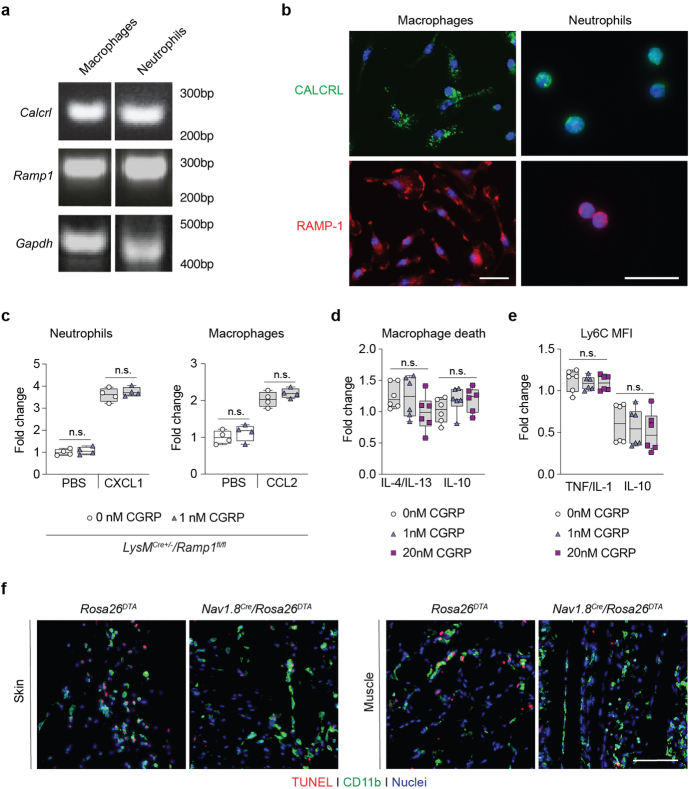
CGRP expression in neutrophils and macrophages, its effects on *Ramp1*^–/–^ cells, M2-Like macrophages, Ly6C expression, and CD11b^+^ cell apoptosis. **a**, *Calcrl* and *Ramp1* expression in bone marrow-derived neutrophils and macrophages detected by RT-PCR. *Gapdh* was used as the housekeeping gene. Repeated independently 3 times. For gel source data, see Supplementary Fig. 1. **b**, CALCRL and RAMP1 expression was detected in bone marrow-derived neutrophils and macrophages using immunostaining. CALCRL, green; RAMP1, red; nuclei, blue. Scale bars = 25 μm. Repeated independently 3 times. **c**, Neutrophils and macrophages derived from mouse bone marrow of *LysMCre*^+/–^/Ramp1^fl/fl^ were treated with saline (PBS, 0 nM CGRP) or CGRP (1 nM) and transwell migration towards a chemoattractant (CXCL1 or CCL2) was tested (*n* = 4). Results are expressed as fold change over the saline PBS/0 nM CGRP control group. **d,e**, Bone marrow-derived macrophages were treated with saline (0 nM CGRP) or CGRP (1 or 20 nM). Cell death in response to CGRP when macrophages were cultured with anti-inflammatory cytokines (IL-4/IL-13, or IL-10) (**d**) (*n* = 6). Ly6C expression in response to CGRP treatment after macrophage culture in inflammatory (TNF/IL-1) or anti-inflammatory conditions (IL-10) (**e**) (*n* = 6). Results are expressed as fold increase over treatment without CGRP and without cytokines. MFI is the geometric-mean of fluorescence intensity. **f**, Representative images of TUNEL assay at D3 post-injury in *Rosa26*^DTA^ and *Nav1.8*^Cre^/Rosa26^DTA^ mice (CD11b, green; TUNEL, red; nuclei, blue). Scale bar = 100 μm. Repeated independently 6 times. All data are plotted in box plots showing median (central line) and IQR (bounds). Whiskers show min. to max. range. Dots represent independent experiments. Two-way ANOVA with Bonferroni *post hoc* test for pair-wise comparisons. n.s., non-significant. Source Data

The increased number of neutrophils and inflammatory monocytes/macrophages observed in the absence of Na_V_1.8^+^ sensory neurons, along with the delayed transition of macrophages towards an anti-inflammatory and pro-repair phenotype, suggested that CGRP may regulate these cells through multiple mechanisms. Thus, we investigated the effect of CGRP on neutrophils and macrophages in vitro. First, we verified that these cells strongly express CGRP receptor subunits (Extended Data Fig. 5a,b). Then, we conducted migration assays and observed that CGRP severely inhibited neutrophil and macrophage migration towards common chemokines found in wounds (CXCL1 for neutrophils and CCL2 for macrophages) (Fig. 2b). Notably, cell migration was also inhibited to some extent in the absence of chemokines. However, CGRP did not inhibit cell migration in *Ramp1*^*−/−*^ neutrophils and macrophages, confirming that CGRP signals via RAMP1–CALCRL to mediate its effects on these cells (Extended Data Fig. 5c). Next, we investigated the effect of CGRP on neutrophil and macrophage viability. CGRP triggered increased neutrophil and macrophage death in the presence of the inflammatory cytokines IL-1β and TNF, which are typically found in acute injuries. Notably, CGRP did not promote macrophage death when cells were cultured without inflammatory cytokines or with cytokines that induce an M2-like polarization, suggesting that CGRP elicits its effect on macrophage death during the inflammatory phase of tissue healing (Fig. 2c and Extended Data Fig. 5d). Subsequently, we explored whether CGRP influences neutrophil clearance by regulating macrophage efferocytosis. Stimulation with CGRP resulted in a marked increase of macrophage efferocytosis, both in the absence and presence of inflammatory cytokines (IL-1β and TNF) (Fig. 2d). Additionally, CGRP had no direct impact on macrophage Ly6C expression in vitro despite our earlier observation of an increased number of pro-inflammatory monocytes/macrophages (Ly6C^hi^) in injured tissues of mice lacking nociceptors (Extended Data Fig. 5e). This suggested that the high number of Ly6C^hi^ monocytes/macrophages was probably a consequence of delayed neutrophil clearance and/or macrophage polarization. Finally, we tested whether CGRP accelerates macrophage polarization towards an M2-like phenotype. We found that in the presence of the typical anti-inflammatory cytokines IL-4 and IL-13 (IL-4/IL-13) or IL-10, CGRP treatment increased levels of the M2-like markers CD206 and arginase-1, respectively, indicating that CGRP accelerates polarization into an anti-inflammatory and pro-repair phenotype (Fig. 2e).

To validate inhibitory migration effects of CGRP in vivo, we used an adoptive transfer model in which tdTomato^+^ bone marrow cells were systemically administered into *Rosa26*^*DTA*^ control and *Nav1.8*^*cre*^*/Rosa26*^*DTA*^ mice following skin and muscle injuries. One day after the transfer, relative accumulation of tdTomato^+^ neutrophils and monocytes/macrophages in the injured tissues was assessed by flow cytometry. Compared with *Rosa26*^*DTA*^ mice, *Nav1.8*^*cre*^*/Rosa26*^*DTA*^ mice showed a significant increase in neutrophils and monocytes/macrophages into injured skin and muscle, suggesting that migration of these cell types into injured tissues was considerably increased (Fig. 2f). For validation of efferocytosis in vivo, we injected dead or dying tdTomato^+^ neutrophils intradermally at the border of skin wounds. The relative number of monocytes/macrophages positive for tdTomato was then assessed by flow cytometry and was found be significantly lower in *Nav1.8*^*cre*^*/Rosa26*^*DTA*^ mice, demonstrating an impairment of efferocytosis (Fig. 2f). Finally, to assess neutrophil and macrophage cell death in vivo, we performed a TUNEL assay on injured tissue sections 3 days post-injury. Slightly fewer CD11b^+^ cells were TUNEL-positive in *Nav1.8*^*cre*^*/Rosa26*^*DTA*^ mice (Fig. 2g and Extended Data Fig. 5f). However, as observed in vitro, the effect on cell death was relatively modest. Thus, CGRP-induced cell death is probably not a major mechanism in vivo. Together, these data support a model in which nociceptor-derived CGRP promotes tissue healing by tightly regulating neutrophil and monocyte/macrophage dynamics, functions and phenotypes in injured tissues, resulting in a faster transition from a pro-inflammatory to a pro-healing phase. CGRP inhibits neutrophil and monocyte/macrophage migration into injured tissues and may further enhance neutrophil and inflammatory macrophage death in the presence of inflammatory cytokines. Meanwhile, CGRP increases neutrophil clearance by stimulating macrophage efferocytosis, which, together with a direct effect of CGRP on macrophage polarization, supports macrophages switching towards an anti-inflammatory and pro-repair phenotype. This model aligns well with the function of neutrophils and macrophages during tissue healing. For instance, excessive mobilization of neutrophils and inflammatory monocytes/macrophages is generally associated with impaired wound healing^1,2,26,31,32^. Enhanced clearance rate of pro-inflammatory cells within injured tissues is also well-known to prevent excessive inflammation and facilitate the transition towards the pro-repair phase. Indeed, impaired tissue healing has been linked to delayed inflammatory cell death and a decrease in macrophage efferocytosis capability^22,33,34^.

## Mediation of the CGRP effect by TSP-1

**Fig. 3:**
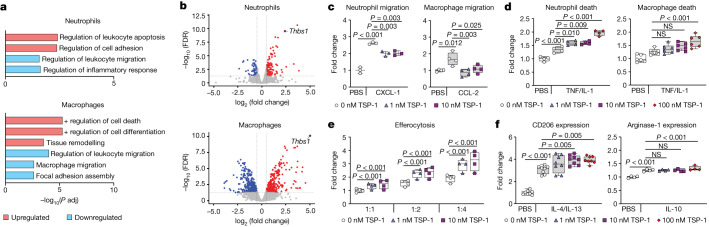
CGRP upregulates TSP-1 in neutrophils and macrophages to mediate its activity. **a**,**b**, RNA-seq analysis of CGRP-treated neutrophils and macrophages (1 nM or saline for 4 h). Differential gene expression was performed with limma-voom (false discovery rate (FDR) < 0.05). Fold change values returned by limma were used for pathway analysis with FDR < 0.05 to correct for multiple comparisons. **a**, GO enrichment analysis of significantly upregulated (red) and downregulated (blue) genes in CGRP-treated and saline-treated groups. **b**, Volcano plots showing DEGs with fold change > |1.5| between CGRP-treated and saline-treated groups. Significantly upregulated (red) and downregulated (blue) genes in CGRP-treated neutrophils and macrophages are shown (*n* = 3). **c**–**f**, Neutrophils and macrophages were treated with saline (PBS, 0 nM TSP-1) or TSP-1 (1, 10 or 100 nM). Results are expressed as fold change over the PBS (0 nM TSP-1) control group. Boxes show median (centre line) and interquartile range (edges), whiskers show the range of values. Dots represent independent experiments. **c**, Transwell migration towards a chemoattractant (CXCL1 or CCL2) after TSP-1 treatment (*n* = 3 for neutrophils, *n* = 4 for macrophages). **d**, Cell death in response to TSP-1 with or without TNF plus IL-1 (*n* = 4). **e**, Macrophage efferocytosis of neutrophils after TSP-1 treatment (*n* = 4). **f**, Macrophage M2-like polarization determined via CD206 and arginase-1 expression in response to TSP-1 and IL-4/IL-13 or IL-10 treatments (CD206, *n* = 8; arginase-1, *n* = 4). **c**–**f**, One-way ANOVA with Tukey post hoc test for pairwise comparisons. *P* values are indicated. Source Data

**Extended Data Fig. 6:**
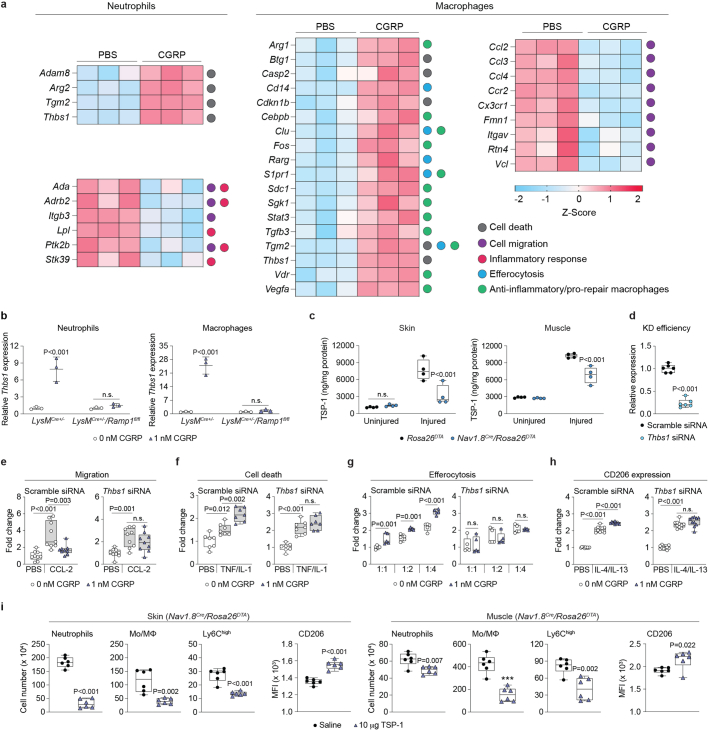
TSP-1 mediates the activity of CGRP on neutrophils and macrophages. **a**, Differentially expressed genes contributing to GO enrichment analysis of CGRP-treated neutrophils and macrophages. Neutrophils and monocytes/macrophages were treated with CGRP (1 nM) or saline for 4 h. Transcriptomic profiling was performed by bulk RNA sequencing. Heatmap of selected significantly upregulated and downregulated genes depicting standardised gene expression values (z-scores) in CGRP-treated cells compared to PBS-treated cells. Individual biological replicates are shown (*n* = 3). Genes are displayed in alphabetical order and further classified according to known functions indicated by the coloured circles next to the heat map. **b**, *Thbs1* expression after CGRP (1 nM) stimulation for 4 h in neutrophils and macrophages isolated form *LysM*^*Cre+/–*^ and *LysM*^*Cre+/–*^/*Ramp1*^*fl/fl*^ mice (*n* = 3). Dots represent independent experiments. Horizontal bars show mean. Whiskers show min. to max. range. **c**, TSP-1 concentration in skin and muscle before (uninjured) and at D3 post-injury quantified by ELISA (*n* = 4). **d**, Knockdown (KD) efficiency of TSP-1 by siRNA verified by qPCR analysis in macrophages. Results are expressed as relative expression over cells transfected with scramble siRNA (n = 6). **e–h**, Macrophages derived from mouse bone marrow were transfected with scramble siRNA or *Thbs1* siRNA and treated with saline (PBS, 0 nM CGRP) or CGRP (1 nM). Results are expressed as fold change over the PBS/0 nM CGRP control group. Transwell migration towards CCL2 in the presence of CGRP (**e**) (*n* = 9). Cell death in response to CGRP with or without TNF/IL-1 (**f**) (*n* = 7). Macrophage efferocytosis of neutrophils after CGRP treatment (**g**) (*n* = 5). Macrophage M2-like polarisztion determined via CD206 expression in response to CGRP and IL-4/IL-13 (**h**) (*n* = 10). **i**, Saline or TSP-1 (total 10 μg) was injected in mouse skin wound borders at D1 and D3 post-injury or delivered in mouse quadriceps volumetric muscle loss defect via a fibrin hydrogel right after injury. Neutrophil and monocyte/macrophage (Mo/Mϕ) populations in injured tissues were analysed by flow cytometry at D3 post-delivery. CD206 level measurements were performed at D14 post-delivery (*n* = 6). MFI is the geometric-mean of fluorescence intensity. All data are plotted in box plots showing median (central line) and IQR (bounds). Whiskers show min. to max. range. Dots represent independent experiments. Two-tailed Student’s *t-*test in **b,c,d,i**. One-way ANOVA with Tukey *post hoc* test for pair-wise comparisons in **e–h**. *P* values are indicated; n.s., non-significant. Source Data

To further understand the molecular mechanisms by which CGRP modulates neutrophils and macrophages, we analysed the transcriptome of bone marrow-derived neutrophils and macrophages after CGRP stimulation by RNA sequencing (RNA-seq). For both cell types, gene ontology (GO) analysis of differentially expressed genes (DEGs) between CGRP-treated and control groups identified biological processes that were congruent with the in vitro effects of CGRP. In addition, the biological processes reflected the differences in neutrophil and macrophage dynamics observed in tissue injuries of control and nociceptor-depleted mice. For instance, for both neutrophils and macrophages, pathways enriched in the upregulated DEGs included cell death, whereas those among the downregulated DEGs included cell migration (for example, *Ccr2*, *Cx3cr1*, *Itgav* and *Itgb3*) (Fig. 3a and Extended Data Fig. 6a). Furthermore, pathways enriched in the upregulated DEGs were associated with tissue remodelling and differentiation in macrophages, featuring genes typically linked to an anti-inflammatory and pro-repair phenotype such as *Arg1*, *Cebpb*, *Stat3*, *Sgk1*, *Tgfb3*, *Tgm2* and *Vegfa*^35^. Finally, in response to CGRP, macrophages upregulated genes that are strongly associated with efferocytosis such as *Cd14*, *Clu*, *S1pr1*, *Rarg* and *Tgm2*^36,37^ (Fig. 3a and Extended Data Fig. 6a). Crucially, we found that thrombospondin-1 (*Thbs1*)—which encodes TSP-1, a multifunctional extracellular matrix (ECM) protein that regulates many biological processes including tissue healing^38^—was the most upregulated gene in both neutrophils and macrophages (Fig. 3b). Further confirming that TSP-1 is expressed in response to CGRP via RAMP1–CALCRL, *Ramp1*^*−/−*^ neutrophils and macrophages did not upregulate *Thbs1* after CGRP stimulation (Extended Data Fig. 6b). Moreover, TSP-1 levels after skin and muscle injuries were lower in *Nav1.8*^*cre*^*/Rosa26*^*DTA*^ mice (Extended Data Fig. 6c).

Notably, it has been reported that macrophages are the primary source of TSP-1 in wounds, and the absence of TSP-1 leads to impaired wound healing and prolonged inflammation^39,40^. Furthermore, increasing evidence suggests that TSP-1 is an important regulator in immune responses^41^. Thus, similar to the assays performed with CGRP, we tested whether TSP-1 affects neutrophil and macrophage migration and death, as well as efferocytosis and macrophage polarization. Although some reports have suggested that TSP-1 promotes neutrophil and macrophage migration^41^, we clearly observed an inhibition of migration when cells were treated with TSP-1, similarly to our observations with CGRP (Fig. 3c). Moreover, TSP-1 accelerated death of neutrophils and macrophages in a dose-dependent manner in the presence of inflammatory cytokines (Fig. 3d). Efferocytosis of neutrophils by macrophages was also greatly enhanced following macrophage treatment with TSP-1 (Fig. 3e), in line with a previous study suggesting that TSP-1 acts as a bridge between neutrophils and macrophages to facilitate efferocytosis^41^. Finally, TSP-1 enhanced expression of CD206 and arginase-1 in the presence of anti-inflammatory cytokines (IL-4/IL-13 or IL-10), demonstrating that TSP-1 accelerates macrophage polarization towards an anti-inflammatory and pro-repair phenotype (Fig. 3f). To further confirm that CGRP exerted its effects primarily via an autocrine or paracrine action of TSP-1, we tested the effect of CGRP upon short interfering RNA (siRNA)-mediated knockdown of *Thbs1* (Extended Data Fig. 6d). Only macrophages were used, as transfection of neutrophils is very challenging. Knockdown of *Thbs1* abolished the effects of CGRP on cell migration, death, efferocytosis and polarization, whereas scramble siRNA showed no effect on all these cellular processes (Extended Data Fig. 6e–h). To validate these effects in vivo, we administered TSP-1 in *Nav1.8*^*cre*^*/Rosa26*^*DTA*^ mice following skin and muscle injuries. TSP-1 delivery resulted in reduced numbers of neutrophils, monocytes/macrophages and inflammatory monocytes/macrophages (Ly6C^hi^) at day 3 post-injury. Additionally, TSP-1 led to an increase in CD206 expression at day 14 post-injury (Extended Data Fig. 6i). Together, these results provide clear support for the role of TSP-1 in mediating the immunomodulatory effect of CGRP on neutrophils and macrophages.

## eCGRP promotes diabetic tissue healing

**Extended Data Fig. 7:**
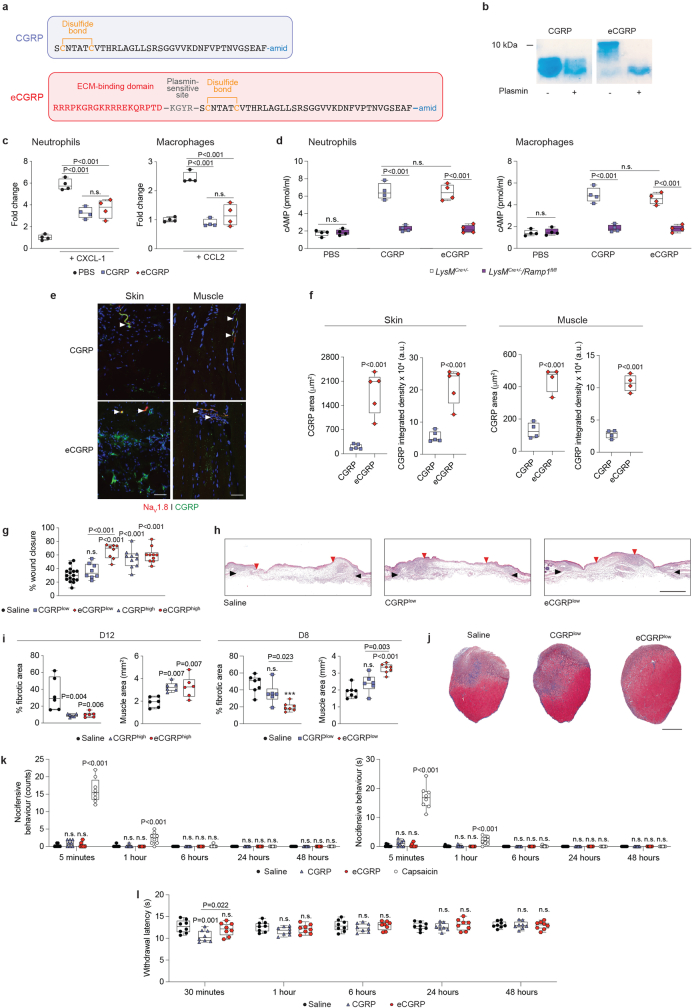
Rescue of tissue healing in *Nav1.8*^*Cre*^/*Rosa26*^*DTA*^ mice by local delivery of CGRP variants. **a**, Design and amino acid sequences of wild-type CGRP and eCGRP. Disulfide bonds are indicated in yellow. Amid indicates amidation of the C-terminus phenylalanine. ECM-binding sequence from placenta growth factor (PlGF) is in red. Plasmin-sensitive site from vitronectin is in grey. **b**, CGRP and eCGRP were incubated with or without plasmin and analysed by SDS-PAGE. The gels show cleavage of the ECM-binding sequence in eCGRP by plasmin. Repeated independently 3 times. For gel source data, see Supplementary Figure 1. **c**, eCGRP activity was assessed using neutrophil and macrophage migration. The graphs show migration towards a chemoattractant (CXCL-1 or CCL2) in the presence of saline (PBS) or CGRP variants (20 nM). Results are expressed as fold change over saline control. Data are plotted in box plots showing median (central line) and IQR (bounds). Whiskers show min. to max. range. Dots represent individual experiments (*n* = 4). **d**, Neutrophils and macrophages were isolated from *LysM*^*Cre+/–*^ and *LysM*^*Cre+/–*^/*Ramp1*^*fl/fl*^ mice. Cells were stimulated with CGRP or eCGRP (1 nM) for 30 min. cAMP concentration in cell lysates was measured by competitive ELISA (*n* = 4). **e,f**, CGRP (1 μg) or equimolar eCGRP was delivered intradermally or intramuscularly in *Nav1.8*^*Cre*^*/Rosa26*^*tdT*^ mice. One day post-delivery, tissues were harvested and CGRP variants were detected by immunostaining. Representative skin and muscle tissue sections. CGRP signal coming from exogenous CGRP variants appears in green. Arrows indicate CGRP signal coming from Na_V_1.8^+^ sensory neurons (in red). Nuclei are in blue. Scale bars = 50 μm. Quantification of CGRP-positive area and signal intensity expressed as integrated density (**f**) (*n* = 5 for skin*, n* = 4 for muscle). **g,h**, Saline, low dose of CGRP (250 ng), high dose of CGRP (500 ng), or equimolar eCGRP was delivered on *Nav1.8*^*Cre*^/*Rosa26*^*DTA*^ mouse skin wounds (D1 post-injury for low dose and D1 and D3 post-injury for high dose). Skin wound closure D6 post-injury evaluated by histomorphometric analysis (**g**) (*n* = 16 for saline; *n* = 8 for low; *n* = 10 for high). Representative skin histology (**h**). Black arrows indicate wound edges and red arrows indicate tips of epithelium tongue. Scale bar = 1 mm. **i,j**, Saline, low dose of CGRP (250 ng), high dose of CGRP (1 μg), or equimolar eCGRP was delivered in *Nav1.8*^*Cre*^/*Rosa26*^*DTA*^ mouse quadriceps volumetric muscle loss defect via a fibrin hydrogel. The extent of muscle regeneration (represented by the percentage of fibrotic tissue and muscle area) was evaluated by histomorphometric analysis of tissue sections at D8 (low dose) and D12 (high dose) post-injury (**i**) (D12: *n* = 6; D8: *n* = 7 for saline and high, *n* = 6 for low). Representative histology (fibrotic tissue is stained dark blue; muscle tissue is stained red) (**j**). Scale bar = 1 mm. **k**, Mice received one injection of saline, CGRP (1 μg), equimolar eCGRP, or capsaicin (positive control) in the right hind paw. Graphs show duration and frequency of nocifensive behaviours recorded for 5 min at various time points (*n* = 8). **l**, Mice received one injection of saline, CGRP (1 μg), or equimolar eCGRP in the right hind paw. Graph shows thermal withdrawal latency at various time points post-injection (*n* = 8). All data are plotted in box plots showing median (central line) and IQR (bounds). Whiskers show min. to max. range. Dots represent independent experiments or injuries. One-way ANOVA with Tukey *post hoc* test for pair-wise comparisons in **c,d,g,i**. Two-tailed Student’s *t*-test in **f**. Two-way ANOVA with Bonferroni *post hoc* test for pair-wise comparisons in **k,l**. *P* values are indicated; n.s., non-significant. Source Data

There is evidence suggesting that CGRP has a role in wound healing. For example, mice lacking CGRP exhibit impaired skin wound healing^42^. Additionally, CGRP application is likely to promote corneal repair^43^. Therefore, since we found that nociceptor-derived CGRP regulates neutrophils and macrophages to facilitate tissue healing, we investigated whether local delivery of CGRP could restore the impaired healing observed in mice ablated of nociceptors. CGRP is a small peptide, which poses a challenge in achieving sustained effects without immediate burst signalling when delivered locally into tissue, as it can rapidly signal to cells, diffuse away from the delivery site and undergo degradation. Moreover, for clinical application, a high concentration of CGRP circulating in the body is undesirable, owing to possible off-target effects^44^. Thus, we engineered CGRP to enhance retention and protection at delivery sites by fusing it to a sequence with a very high affinity for ECM components^25,26,45^. The ECM-binding sequence was fused to the N terminus of CGRP followed by a plasmin-sensitive sequence to allow the release of CGRP from ECM via proteolytic activity (Extended Data Fig. 7a,b). These modifications did not impair CGRP activity, as demonstrated by the preserved capacity of the engineered CGRP (eCGRP) to inhibit neutrophil and macrophage migration and induce cAMP in these cells via RAMP1–CALCRL (Extended Data Fig. 7c,d). As demonstrated previously^25,26,45^, the ECM binding enabled better retention of CGRP after delivery into tissues (Extended Data Fig. 7e,f). We then tested whether CGRP variants (wild-type CGRP and eCGRP) could promote closure of splinted skin wounds and the regeneration of quadriceps after volumetric muscle loss in *Nav1.8*^*cre+/−*^/*Rosa26*^*DTA*^ mice. For skin, CGRP variants were delivered topically, whereas for muscle, they were delivered via a fibrin hydrogel. Both CGRP variants enhanced the extent, compared with saline control, of wound closure and muscle regeneration upon delivery of 1 μg. Moreover, at a lower dose (250 ng) eCGRP promoted greater wound closure and muscle regeneration compared with wild-type CGRP (Extended Data Fig. 7g–j). Notably, delivering a relatively high dose of CGRP (10 μg) into mice has been shown to contribute to peripheral nociceptive sensitization^46^. Thus we tested whether injection of CGRP variants induced pain (Extended Data Fig. 7k,l). Administration of wild-type CGRP or eCGRP into mouse hind paws did not elicit significant pain behaviours over the 48-h experiment. However, a transient and slight increase in thermal sensitivity was observed only for wild-type CGRP. This difference is probably owing the binding of eCGRP to the ECM, which prevents an immediate burst of signalling after delivery^25,26,45^.

**Fig. 4:**
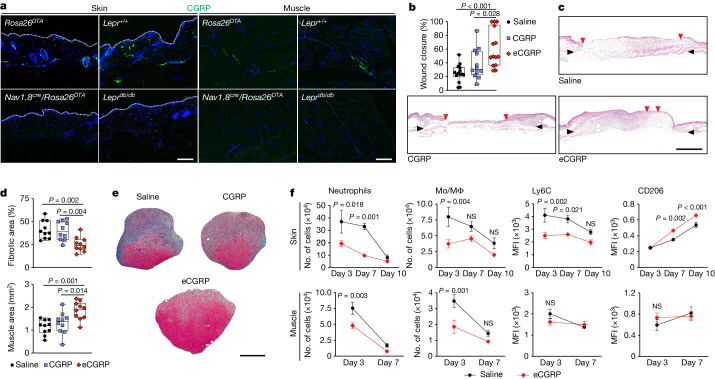
Delivery of eCGRP promotes tissue healing in diabetic mice. **a**, CGRP expression in skin and muscle of *Nav1.8*^*cre*^*/Rosa26*^*DTA*^ and diabetic (*Lepr*^*db/db*^) mice detected by immunostaining. CGRP, green; nuclei, blue. White lines indicate the keratinocyte layer. Scale bars, 300 μm. The experiment was repeated independently 6 times. **b**,**c**, Saline, CGRP (500 ng) or equimolar eCGRP was delivered on *Lepr*^*db/db*^ skin wounds at day 1 and day 3 post-injury. **b**, Wound closure was evaluated by histomorphometric analysis on day 10 post-injury (saline and eCGRP, *n* = 14; CGRP, *n* = 12). Boxes show median (centre line) and interquartile range (edges), whiskers show the range of values. **c**, Representative skin histology. Black arrows indicate wound edges and red arrows point to tips of epithelium tongue. Scale bar, 1 mm. **d**,**e**, Saline, CGRP (1 μg) or equimolar eCGRP was delivered in *Lepr*^*db/db*^ quadricep volumetric muscle loss defect via a fibrin hydrogel. **d**, Muscle regeneration (represented by the percentage of fibrotic tissue and muscle area) was evaluated by histomorphometric analysis on day 8 post-injury. Boxes show median (centre line) and interquartile range (edges), whiskers show the range of values (*n* = 10). **e**, Representative histology (fibrotic tissue is stained blue; muscle tissue is stained red). Scale bar, 500 μm. **f**, Numbers of neutrophils (CD11b^+^Ly6G^+^F4/80^–^) and monocytes/macrophages (CD11b^+^F4/80^+^Ly6G^–^), and expression of Ly6C and CD206 in macrophages (represented by MFI) in *Lepr*^*db/db*^ skin wounds and muscle injuries treated with saline or eCGRP were quantified by flow cytometry. Skin: *n* = 6. Muscle: day 3, *n* = 8 for saline, *n* = 7 for eCGRP; day 7, *n* = 9. Data are plotted as kinetic line plots showing mean ± s.e.m. **b**,**d**, One-way ANOVA with Tukey post hoc test for pairwise comparisons. **f**, Two-way ANOVA with Bonferroni post hoc test for pairwise comparisons. *P* values are indicated. Source Data

**Extended Data Fig. 8:**
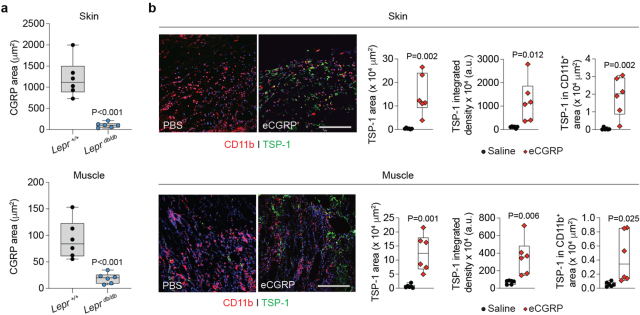
CGRP levels in *Lepr*^*db/db*^ mice and TSP-1 deposition in response to eCGRP delivery. **a**, CGRP expression in uninjured skin and muscle of wildtype (*Lepr*^*+/+*^) and diabetic (*Lepr*^*db/db*^) mice was detected by immunostaining of tissue sections. The graphs show quantification of CGRP-positive area (*n* = 6). **b**, Skin wounds and muscle defects in *Lepr*^*db/db*^ mice were treated with eCGRP. Expression of TSP-1 in granulation tissue was detected at D4 via immunostaining of tissue sections. TSP-1 in green, myeloid cells (CD11b) in red, nuclei in blue. Scale bar = 200 μm. Graphs show quantification of TSP-1–positive area, TSP-1 signal intensity expressed as integrated density and TSP-1-positive area in CD11b^+^ cells (*n* = 6). All data are plotted in box plots showing median (central line) and IQR (bounds). Whiskers show min. to max. range. Dots represent individual tissue sections. Two-tailed Student’s *t-*test in **a,b**. *P* values are indicated. Source Data

**Extended Data Fig. 9:**
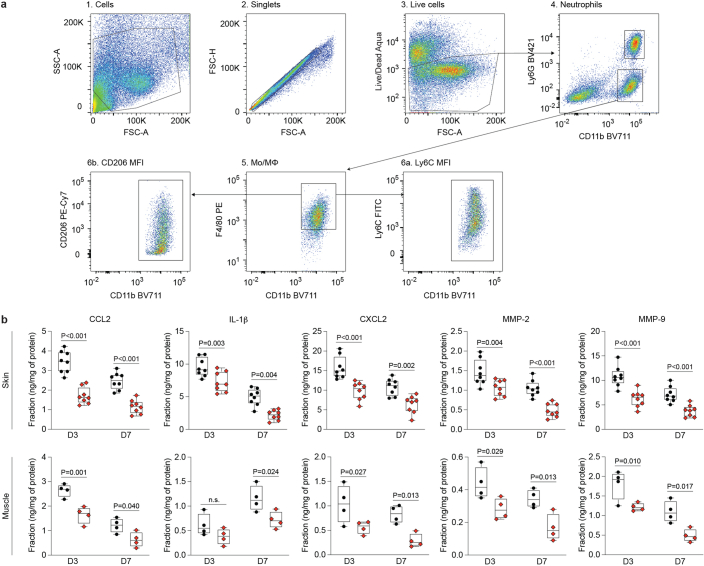
Gating strategy to analyse wound immune cell dynamics in diabetic mice as well as cytokine and protease levels after eCGRP treatment. **a**, Gating strategy to analyse myeloid cells skin and muscle post-injury. Flow cytometry dot plots representing the step by step (1–6) gating strategy to identify neutrophil (CD11b^+^, Ly6G^+^, F4/80^–^) and macrophage populations (CD11b^+^, F4/80^+,^ Ly6G^–^). MFI is the geometric-mean of fluorescence intensity. **b**, Saline or eCGRP was delivered on *Lepr*^*db/db*^ skin wounds or in quadricep volumetric muscle loss defect via a fibrin hydrogel. The levels of CCL2, IL-1β, CXCL2, MMP-2, and MMP-9 in injured tissues were quantified by ELISA (*n* = 8 for skin, *n* = 4 for muscle). Data are plotted in box plots showing median (central line) and IQR (bounds). Whiskers show min. to max. range. Dots represent individual injuries. Two-way ANOVA with Bonferroni *post hoc* test for pair-wise comparisons. *P* values are indicated. Source Data

We next searched for a model with more clinical relevance compared with the *Nav1.8*^*cre+/−*^/*Rosa26*^*DTA*^ mice. Indeed, more than half of patients with diabetes develop peripheral neuropathy, characterized by the presence of dysfunctional peripheral nerves and a decrease in intraepidermal nerve fibres^47,48^. Consequently, the decrease in neuropeptide levels, including CGRP, may disrupt neuro–immune interactions crucial for tissue healing^49,50^. Indeed, patients with diabetes commonly experience chronic non-healing wounds, which represent the most prevalent and severe complication of the condition, alongside the development of muscle atrophy^49–51^. The diabetic *Lepr*^*db/db*^ mouse is a model for type 2 diabetes that is commonly used to study impaired tissue healing, since it mimics some aspects of human chronic wounds, including immune dysregulation and peripheral neuropathies^25,26,52^. Additionally, it has been shown that *Lepr*^*db/db*^ have impaired muscle regeneration^53^. Thus, we investigated the distribution of CGRP in *Lepr*^*db/db*^ mice skin and muscle to confirm that these mice exhibited peripheral neuropathy. We observed a significant reduction in neuron-like structures expressing CGRP in both skin and muscle of *Lepr*^*db/db*^ mice, mirroring the pattern seen in *Nav1.8*^*cre+/−*^/*Rosa26*^*DTA*^ mice (Fig. 4a and Extended Data Fig. 8a). This observation supported the use of diabetic mice to assess the regenerative potential of local CGRP delivery. Notably, wound closure and muscle regeneration was greatly improved when injuries were treated with eCGRP, compared with saline control and wild-type CGRP (Fig. 4b–e). We also examined TSP-1 expression in granulation tissue post-eCGRP delivery, given the induction of TSP-1 expression by CGRP in neutrophils and macrophages. Immunostaining revealed a significant increase in TSP-1 deposition, colocalizing to some extent with myeloid (CD11b^+^) cells in skin and muscle granulation tissues (Extended Data Fig. 8b). Next, since neutrophil and macrophage dynamics were disrupted in *Nav1.8*^*cre+/−*^/*Rosa26*^*DTA*^ mouse injuries, we investigated whether eCGRP delivery modulated those cells in injured tissues (Extended Data Fig. 9). eCGRP delivery reduced the number of neutrophils and monocytes/macrophages in both skin and muscle injures at early timepoints post-injury. Moreover, eCGRP delivery led to reduced levels of the pro-inflammatory marker Ly6C and increased the level of the anti-inflammatory marker CD206 in skin wounds (Fig. 4f). Indeed, high numbers of neutrophils and inflammatory macrophages in diabetic injured tissues are known to delay healing^2,31,32^. An abnormally high number of neutrophils in chronic wounds leads to an over-production of pro-inflammatory cytokines, reactive oxygen species and proteases^54^, and increased NETosis^55^. Similarly, failure to convert macrophages to an anti-inflammatory phenotype leads to high levels of pro-inflammatory cytokines and proteases^32^. Thus, we measured concentrations of inflammatory cytokines/chemokines and proteases in response to saline and eCGRP treatment. eCGRP resulted in a significant reduction of IL-1β, CCL2, CXCL2, MMP-2 and MMP-9 (Extended Data Fig. 9b)—factors that are known to impair tissue healing when their levels are increased^26,56,57^. Thus, the results overall support the idea that the local delivery of eCGRP accelerates the transition of diabetic injured tissues towards an anti-inflammatory and pro-repair phase.

## Conclusion

**Extended Data Fig. 10:**
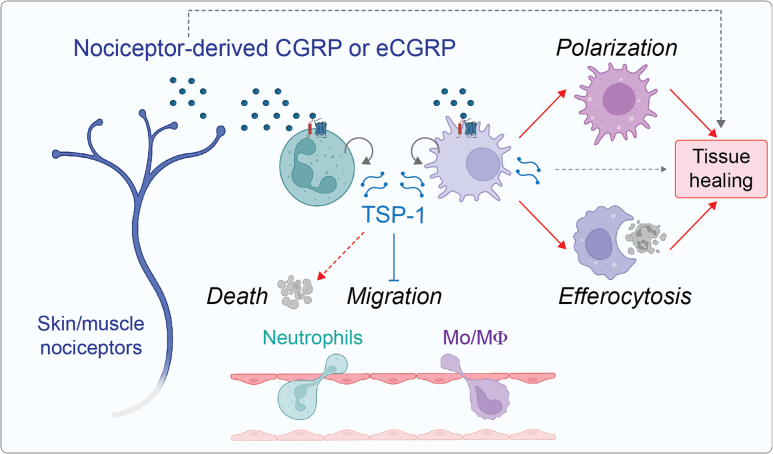
Summary of the neuro-immune-regenerative axis after acute injury in skin and muscle. The schematic shows the proposed mechanisms by which nociceptors promote tissue healing via controlling neutrophils and monocytes/macrophages (Mo/Mϕ) in injured tissues. Following tissue injury, CGRP-expressing nociceptor endings grow into the granulation tissue. CGRP signalling in neutrophils and macrophages induces the release of the ECM protein TSP-1. TSP-1 is deposited in the injured tissue milieu inhibiting neutrophil and monocytes/macrophage migration and eventually accelerating the cell death response of neutrophils and macrophages to inflammatory cytokines. In addition, CGRP promotes efferocytosis and macrophage polarization into a M2-like phenotype via an autocrine or paracrine effect of TSP-1. Overall, nociceptors are critical for the transition of the injured tissue microenvironment towards a tissue healing phase. Blue line indicates inhibition, red line indicates induction. Dashed grey lines indicate that CGRP and TSP-1 may also promote tissue healing by acting on non-immune cells. Created with BioRender.com.

CGRP and nociceptors have been shown to have immunomodulatory effects in various contexts^7–12^. For example, CGRP has a key role as an immunosuppressive mediator during sepsis^58^. Additionally, during bacterial infection, nociceptor-derived CGRP has been shown to induce an anti-inflammatory transcriptional programme in macrophages and suppress neutrophil recruitment^12^. However, the role of nociceptors during tissue healing after acute injury was unclear. This study reveals the existence of an important neuro–immune regenerative axis following acute tissue injury and highlights the intricate interplay between nociceptors, immune cells and tissue healing processes. We found that Na_V_1.8^+^ sensory neurons expressing CGRP, which mostly represent nociceptors, extend into the granulation tissue formed after skin and muscle injuries and profoundly modulate neutrophil and macrophage dynamics during tissue healing. This neuro–immune modulation promotes the transition towards an anti-inflammatory and pro-repair phase, which ultimately facilitates the healing process. Mechanistically, we demonstrate that CGRP signalling in neutrophils and macrophages induces the expression of TSP-1, which in turn limits their accumulation and accelerates cell death in the presence of inflammatory cytokines. In addition, CGRP promotes neutrophil clearance via efferocytosis and boosts macrophage polarization into an anti-inflammatory and pro-repair phenotype via autocrine and paracrine effects of TSP-1 (Extended Data Fig. 10). Collectively, the mechanisms uncover important implications for advancing our understanding of the tissue healing process after acute injury. These findings also have significant implications for advancing regenerative medicine, particularly for patients with peripheral neuropathies, including those associated with conditions such as diabetes. Harnessing the potential of this neuro–immune regenerative axis opens new avenues for effective therapies, whether as standalone treatments or in combination with existing therapeutic approaches. Utilizing or mimicking neuro–immune interactions holds great promise for addressing chronic wounds and other non-healing tissues, in which dysregulated neuro–immune interactions have a pathologic role and impair tissue healing.

## Methods

### Ethical statement for animal experiments

Animal experiments were approved by the Monash Animal Research Platform ethics committee and the Animal Research Committee of the Research Institute for Microbial Diseases of Osaka University (approval numbers 13294, 13335, 17075, 14013 and 23006).

### Animals

Wild-type C57BL/6 J mice were from the Monash Animal Research Platform. Sperm from *Nav1.8*^*cre+/+*^ mice (B6.129-*Scn10a*^*tm2(cre)Jnw/*^H B6, stock ID EM:04582, European Mouse Mutant Archive) were used for in vitro fertilization to generate *Nav1.8*^*cre+/−*^ mice on a C57BL/6 J background. *Rosa26*^*DTA+/+*^ mice (B6.129-*Gt(ROSA)26Sor*^*tm1(DTA)Mrc*^/J), strain 010527, Jackson Laboratory) were maintained on a C57BL/6 J background. To delete sensory neurons expressing Na_V_1.8, *Nav1.8*^*cre+/−*^ mice were bred with *Rosa26*^*DTA+/+*^ mice to generate *Nav1.8*^*cre+/−*^*/Rosa26*^*DTA+/−*^ mice. *Nav1.8*^*cre−/−*^*/Rosa26*^*DTA+/−*^ littermates were used as controls. For visualizing Na_V_1.8^+^ neurons, *Rosa26*^*tdT*^ reporter mice (B6.Cg-*Gt(ROSA)26Sor*^*tm14(CAG-tdTomato)Hze*^/J, strain 007914, Jackson Laboratory) were bred with *Nav1.8*^cre+/+^ mice to generate *Nav1.8*^*cre*^^*+/−*^*/Rosa26*^*tdT+/−*^. *Lepr*^*db/db*^ mice (BKS.Cg-*Dock7*^*m*^ + */+Lepr*^*db*^/J, strain 000642) were obtained from Jackson Laboratory. Mice were bred as heterozygotes to generate *Lepr*^*db/db*^ and *Lepr*^*db/+*^ littermates. B6.129S2-*Ramp1*^*<tm1.2Tsuj>*^ mouse sperm was kindly provided K. Tsujikawa and used for in vitro fertilization to generate *Ramp1*^*fl/+*^ mice. *Ramp1*^*−/−*^ mice were generated by crossing *Ramp1*^*fl/fl*^ mice with *CAG*^*cre*^ mice (C57BL/6-Tg^*(CAG-cre)13Miya*^, RIKEN BioResource Research Center, strain 09807). Specific deletion of *Ramp1* in myeloid cells (*LysM*^*cre*^^*+/−*^*/Ramp1*^*fl/fl*^ mouse) was done by crossing *Ramp1*^*fl/fl*^ mice with *LysM*^*cre*^^*+/+*^ mice (B6.129P2-*Lyzs*^*<tm1(cre)Ifo>*^, RIKEN BioResource Research Center, strain 02302). *LysM*^*cre*^^*+/−*^ littermates were used as controls. To obtain mice constitutively expressing tdTomato, *Rosa26*^*tdT*^ mice were crossed with B6.C-Tg(CMV-cre)1Cgn/J mice from Jackson Laboratory (strain 006054).

### Full-thickness skin wound model

Male 10- to 12-week-old mice were used for most experiments, except for experiment with CGRP variant delivery in which female (10- to 12-week-old non-diabetic or 12- to 14-week-old diabetic) mice were used. Full-thickness punch-biopsy wounds (5 mm in diameter) were created while mice were under isoflurane anaesthesia as described^25,26^. For analgesia, mice received subcutaneous administration of 0.1 mg kg^−1^ buprenorphine. In non-diabetic mice, wounds were covered with a round seal spot plaster (22.5 mm, Livingstone International, Australia) secured with 3M surgical tape. In experiments involving CGRP delivery, a nylon ring (Zenith 5/16 inch and M8 Nylon Washer) was attached with superglue (UHU) to prevent wound contraction. Wounds received topical treatment with either 10 μl of saline (PBS) or a CGRP variant in PBS. Solutions were applied in two different dosages: 250 ng of CGRP or equimolar eCGRP on day 1 post-injury for the low dose, and 500 ng of CGRP or equimolar eCGRP on day 1 and day 4 post-injury for the high dose. For diabetic mice, 4 wounds were created and treated with PBS or CGRP variant in PBS (500 ng CGRP or equimolar eCGRP) on day 1 and day 3.

### Volumetric muscle loss model

Non-diabetic (10- to 12-week-old) and diabetic (12- to 14-week-old) male mice underwent isoflurane anaesthesia. For analgesia, mice received subcutaneous administration of 0.1 mg kg^−1^ buprenorphine. A 1-cm unilateral incision was made, exposing the fascia. Muscle injuries were created either with a 3-mm biopsy punch or by excising a 3 mm × 5 mm segment of the quadriceps, including the rectus femoris muscle. In experiments involving CGRP delivery, muscle defects were covered with a fibrin matrix (40 μl total, 8 mg ml^−1^ fibrinogen (Enzyme Research Laboratories), 12 U ml^−1^ bovine thrombin (Sigma), 5 mM CaCl_2_, and 17 μg ml^−1^ aprotinin (Roche, Sigma)) containing CGRP (250 ng or 1 μg for non-diabetic mice and 1 μg for diabetic mice) or equimolar eCGRP. The incision site was sutured with non-absorbable sutures.

### Adoptive transfer of bone marrow cells

Bone marrow cells (1 × 10^7^) from 6-week-old *Ramp1*^*−/−*^ or wild-type C57BL/6 J mice were intravenously injected into lethally irradiated 6-week-old recipient wild-type or *Ramp1*^*−/−*^ mice that received 100 mg l^−1^ neomycin sulfate for 2 weeks post-irradiation. Skin or muscle defect surgeries were performed 6 weeks after transplantation.

### Histological analysis

Skin wounds were collected using an 8-mm biopsy punch, fixed in 10% formalin at room temperature for 24 h, cut at the edge of the wounds, embedded in paraffin and sectioned at 4 μm until the centre of the wound was passed. Re-epithelialization was measured by histomorphometric analysis. Slides were stained with haematoxylin and eosin, and the centre of the wound was determined by measuring the distance between the panniculus carnosus muscle gap using Aperio ImageScope Viewer (Leica Biosystems). Closure was calculated as the ratio of epidermis closure to the length of the panniculus carnosus gap. Muscle injury sites, including the proximal and distal quadriceps segments, were collected, fixed in 10% formalin solution for 24 h, embedded in paraffin, and sectioned at 4 μm thickness for 5 depths, starting from the edge of the patella, passing the centre of the wound, up to the proximal end of the defect site. Cross-sections were stained with Masson’s Trichrome. Muscle regeneration was determined by averaging the percentage of blue-stained fibrotic area (normalized to the total area) and the remaining non-fibrotic muscle area across five tissue section depths, using Aperio ImageScope.

### Immunohistochemistry for neuropeptides, TSP-1 and myeloid cells

Tissues and DRGs (L1–L6 vertebrae) were fixed in 4% paraformaldehyde, cryoprotected in 30% sucrose, and embedded in OCT compound for 10 μm cryosections. Sections were stored at −20 °C, thawed, permeabilized and blocked with 1% bovine serum albumin (BSA), 10% normal goat serum (NGS) or normal donkey serum in PBS for 1 h. Sudan Black B solution (0.1% in 70% ethanol) was applied for 10 min. For neuropeptide detection, primary antibodies were added in staining buffer (0.5% BSA, 5% NGS or 0.5% BSA, 5% normal donkey serum in PBS) overnight at 4 °C. The primary antibodies included rabbit anti-CGRP (66.7 μg ml^−1^, Sigma, C8198), rabbit anti-substance P (1:500, Thermo Fisher Scientific, 20064), rabbit anti-VIP (1:500, Thermo Fisher Scientific, 20077), and goat anti-galanin (1 μg ml^−1^, Abcam, 99452). For TSP-1 detection, sections were incubated with AffiniPure Fab Fragment goat anti-mouse IgG (H + L) at 100 μg ml^−1^ (Jackson ImmunoResearch Labs, 115-007-003) in PBS for 2 h at room temperature, followed by mouse anti-thrombospondin-1 (5 μg ml^−1^, Thermo Fisher Scientific, 14-9756-82). For myeloid cell detection, slides were incubated with rat anti-mouse CD11b (5 μg ml^−1^, Thermo Fisher Scientific, 14-0112-82). Sections were washed and incubated with respective secondary antibodies for 1 h at room temperature. The secondary antibodies included F(ab′)2-Goat anti-Rabbit IgG Alexa Fluor 488 (2.6 μg ml^−1^, Thermo Fisher Scientific, A-11070), donkey anti-goat IgG Alexa Fluor 488 (2.6 μg ml^−1^, Thermo Fisher Scientific, A-11055), goat anti-mouse IgG Alexa Fluor Plus 488 (2.6 μg ml^−1^, Thermo Fisher Scientific, A48286TR), and goat anti-rat IgG Alexa Fluor Plus 594 (2.6 μg ml^−1^, Thermo Fisher Scientific, A48264). Counterstaining with DAPI for 10 min and mounting with Fluoroshield followed. Imaging was done using Leica DMi8 fluorescent microscope and Leica SP8 inverted confocal microscope.

### Evaluation of neuropeptide expression

Skin and muscle samples from male *Nav1.8*^*cre*^^*+/−*^*/Rosa26*^*tdT+/−*^ mice (10- to 12-week-old) were immunostained as detailed above, imaged on a Leica DMi8 fluorescent microscope and processed using Fiji^59^. Binary images were created with an optimal threshold, and overlapping areas were determined by combining region of interest binary images. Area fraction values, indicating neuropeptide expression in Na_V_1.8^+^ nerves, were calculated based on pixel ratios and converted using a built-in scale bar^60,61^.

### Immunofluorescence for Ki-67 and KRT14

Paraffin sections underwent 20-minute antigen retrieval in 10 mM sodium citrate buffer (pH 6.0), followed by PBS washes and 5-minute permeabilization (0.2% Triton X-100 in PBS). Blocking with 10% NGS in 1% BSA/PBS occurred for 2 h, and endogenous IgG was blocked with unconjugated affinity-purified F(ab) fragment anti-mouse IgG (H + L) (10 μg ml^−1^, Jackson ImmunoResearch, AB_2338476) for 1 h at room temperature. Staining overnight at 4 °C utilized rat anti-mouse Ki-67 (5 μg ml^−1^, Thermo Fisher Scientific, 5698-82) and mouse anti-mouse cytokeratin 14 (4 μg ml^−1^, Thermo Fisher Scientific, MA5-11599) in 1% NGS in PBS with 0.1% BSA. After PBS-T washes, incubation with secondary antibodies occurred: goat anti-mouse Alexa Fluor 647 (2 μg ml^−1^, Thermo Fisher Scientific, A-21235) and goat anti-rat Alexa Fluor 488 (2.67 μg ml^−1^, Thermo Fisher Scientific, A48262TR) for 1 h at room temperature, followed by PBS-T wash. Counterstaining with DAPI (1 μg ml^−1^) for 10 min at room temperature preceded mounting with Fluoroshield.

### TUNEL assay

The In Situ Cell Death Detection Kit, TMR red (Roche, 12156792910) was used, following the manufacturer’s instructions on muscle and skin tissue cryosections. To detect CD11b^+^ cells, sections were incubated overnight at 4 °C with rat anti-mouse CD11b (5 μg ml^−1^, M1/70, Thermo Fisher Scientific, 14-0112-82) in staining buffer. After PBS-T washes, sections were incubated with Alexa Fluor 488 goat anti-rat antibody (2.67 µg ml^−1^, Thermo Fisher Scientific, A48262TR), washed with PBS-T, and counterstained with DAPI (1 μg ml^−1^) before mounting with Fluoroshield. Two tissue section levels were evaluated per sample to determine the percentage of TUNEL^+^ apoptotic cells over total CD11b^+^ cells, examining three fields per section within the injury site.

### Fibroblast, keratinocytes, myoblasts and endothelial cell maintenance

Human umbilical vein endothelial cells (HUVECs; Sigma, 200P-05N) cultured in EGM-2 medium (Lonza, CC-4176) up to 3 passages, and primary mouse fibroblasts from C57BL/6 J mouse tails^26^ (passages 2–3) were used. MCDB-131 medium (Thermo Fisher Scientific) with 100 mg ml^−1^ penicillin/streptomycin and 2 mM glutamine was employed for proliferation assays. C2C12 mouse myoblasts (CellBank Australia) were cultured in a 1:1 ratio of DMEM to F10 medium (2 mM glutamine, 10% FBS, 100 units ml^−1^ penicillin/streptomycin). HaCaT keratinocytes (a gift from R. Boyd) were cultured in DMEM without Ca^2+^ and Mg^2+^ (2 nM glutamine, 10% Chelex-treated FBS, 0.03 nM calcium chloride, 100 units ml^−1^ penicillin/streptomycin) for at least 3 passages. Cells obtained from vendors were authenticated and certified negative for *Mycoplasma* contamination. For proliferation assays, FBS was reduced to 2% or kept at 10% for 24 h. Detached with TrypLE, cells were seeded (2,000 cells per well for HUVECs, fibroblasts, HaCaTs; 1,000 cells per well for C2C12) and treated with CGRP (1 or 20 nM) or 10–20% FBS. Incubation for 48 h (fibroblasts, C2C12) or 72 h (HUVECs, HaCaTs) at 37 °C with 5% CO_2_ followed. Proliferation was determined using the CyQUANT Cell Proliferation Assay (Invitrogen), presented as fold change over basal proliferation (medium only). PerkinElmer EnVision multi-mode plate reader with EnSpire Manager software was used.

### Flow cytometry with tissue samples

Skin wounds were collected using an 8-mm biopsy punch, and muscle defects were dissected to isolate the quadriceps. Samples were minced with scissors and subjected to two serial digestions with collagenase XI (1 mg ml^−1^) at 37 °C (two times 20 min for skin, two times 15 min for muscle). After the first digestion, the supernatant was collected and mixed with neutralization buffer (DMEM/F12 with 10% FBS and 5 mM EDTA). The first collection was kept on ice and fresh collagenase XI was added to the undigested tissue for the second digestion. Digestion mixtures were passed through a 70-μm cell strainer and stained with LIVE/DEAD Fixable Aqua dye (Thermo Fisher Scientific, 1:400 dilution in PBS) for 20 min on ice. Cells were incubated with TruStain FcX anti-CD16/32 (10 μg ml^−1^; clone 93, BioLegend) diluted in staining buffer (5% FBS and 2 mM EDTA in PBS) for 20 min and subsequently incubated with primary antibodies in staining buffer for a further 30 min on ice. The following anti-mouse antibodies from BioLegend were used: FITC anti-CD11b (clone M1/70, 6.6 μg ml^−1^) or BV711 anti-CD11b (clone M1/70, 2 μg ml^−1^); PE anti-F4/80 (clone BM8, 4 μg ml^−1^); BV421 anti-Ly6G (clone 1A8, 2 μg ml^−1^); BV711 anti-Ly6C (clone HK1.4, 1 μg ml^−1^) or FITC anti-Ly6C (clone HK1.4, 5 μg ml^−1^); PE-Cyanine7 anti-CD206 (clone C068C2, 2.6 μg ml^−1^); APC anti-CD206 (clone C068C2, 2 μg ml^−^^1^); PE-Cyanine7 anti-CD3 (clone 17A2, 4 μg ml^−1^); BV711 anti-CD3 (clone 17A2, 4 μg ml^−1^);APC anti-CD4 (clone GK1.5, 2 μg ml^−1^); BV421 anti-CD8 (clone 53-6.7, 2 μg ml^−1^); PE anti-TCR β (clone H57-597, 2 μg ml^−1^); APC/Fire 750 anti-TCR γ/δ (clone GL3, 2 μg ml^−1^); PE-Cyanine anti-CD11c (clone N418, 2 μg ml^−1^); APC/Fire 750 anti-MHC Class II (clone M5/114.15.2, 2 μg ml^−1^). Cells were washed once with a large volume of staining buffer before analysis with BD LSR Fortessa X-20 and FlowJo software (BD Biosciences).

### Mouse bone marrow neutrophil and monocyte isolation

Bone marrow cells were flushed from femora and tibiae of C57BL/6 J mice (8- to 12-week-old) with HBSS without Ca^2+^ and Mg^2+^ containing 2% FBS and 1 mM EDTA. Cell suspension was passed through a 70-μm strainer. Next, EasySep Mouse Neutrophil Enrichment Kit or EasySep Mouse Monocyte Isolation Kit (STEMCELL Technologies) was used to isolate neutrophils or monocytes according to the manufacturer’s instructions. Neutrophils were resuspended in RPMI containing 100 units ml^−1^ penicillin/streptomycin and 10% FBS for cell migration assay and cell death assay or 2% FBS for efferocytosis. Monocytes were cultured in DMEM/F12 (Thermo Fisher Scientific) containing 10% FBS, 2–10 ng ml^−1^ M-CSF (PeproTech) and 100 units ml^−1^ penicillin/streptomycin for subsequent experiments. RAMP1 and CALCRL were detected on neutrophils and monocytes using rabbit anti-RAMP1 (8.5 μg ml^−1^, Alomone Lab, ARR-021) and rabbit anti-calcitonin receptor-like receptor (5 μg ml^−1^, Biorbyt, orb526584).

### Neutrophil cell death

Bone marrow-isolated neutrophils were cultured in RPMI 1640 medium (10% FBS). Cells were incubated with CGRP (1-20 nM, Tocris Bioscience, 83651-90-5) for 10 min, followed by treatment with IL-1 (5 ng ml^−1^) and TNF (50 ng ml^−1^) for 12 h at 37 °C with 5% CO_2_ to induce cell death. After 12 h, cells were washed with PBS and incubated with LIVE/DEAD Fixable Aqua dye (Thermo Fisher Scientific, 1:400 dilution) in PBS on ice for 20 min. Cell death was assessed using BD LSR Fortessa X-20 and FlowJo software (BD Biosciences).

### Macrophage cell death and polarization marker expression

Bone marrow cells from 8- to 12-week-old C57BL/6 J mice were flushed, filtered and cultured in conditioned medium (DMEM/F12 with 10% heat-inactivated FBS, 100 units ml^−1^ penicillin/streptomycin, and 20% L929 fibroblasts-conditioned medium) at 37 °C with 5% CO_2_. After 7–9 days, differentiated macrophages were collected and seeded in 12-well or 6-well plates. The next day, cells were treated with CGRP (1 or 20 nM, Tocris Bioscience, 83651-90-5) for 20 min before exposure to mouse IL-1 (5 ng ml^−1^) and TNF (50 ng ml^−1^), IL-4 (2 ng ml^−1^) and IL-13 (2 ng ml^−1^), or IL-10 (2 ng ml^−1^) (PeproTech Inc) for 24 or 72 h. Macrophages were detached with TrypLE (Gibco) containing 3 mM EDTA, stained with LIVE/DEAD Aqua dye for 20 min on ice, and incubated with blocking solution (10 μg ml^−1^ TruStain FcX anti-CD16/32 (clone 93, BioLegend)) for 20 min before staining with antibodies for 30 min on ice. Antibodies from BioLegend included PE anti-CD11b (clone M1/70, 1 μg ml^−1^), BV711 anti-F4/80 (clone BM8, 2 μg ml^−1^), APC anti-CD80 (clone 16-10A1, 0.5 μg ml^−1^) and PE-Cyanine7 anti-CD206 (clone C068C2, 1 μg ml^−1^). For intracellular staining, cells were fixed and permeabilized using FluoroFix Buffer and Intracellular Staining Permeabilization Wash Buffer (Perm buffer, BioLegend). APC anti-mouse arginase-1 (Thermo Fisher, Clone AlexF5, 1 μg ml^−1^) was added to the Perm buffer and incubated with the cells for 30 min on ice. After washing with Perm buffer and staining buffer, cells were analysed using BD LSR Fortessa X-20 and FlowJo software (BD Biosciences).

### Neutrophil and macrophage migration

Assays were conducted using 6.5-mm-diameter culture plate inserts (Corning) with 5-μm and 3-μm pore sizes for macrophages and neutrophils, respectively. Macrophages (1 × 10^5^) or neutrophils (3 × 10^5^) in migration media (DMEM/F12 with 0.25% BSA) were added to the inserts. The lower chambers contained migration buffer alone or chemoattractant (mouse CCL2 10 ng ml^−1^ for macrophages or mouse CXCL1/KC 150 ng ml^−1^ for neutrophils, PeproTech) with or without CGRP. Cells were allowed to migrate through the insert membrane for 3-4 h at 37 °C with 5% CO_2_. For macrophages, the inserts were then fixed with 4% paraformaldehyde, and cells on the upper side were removed. DAPI (1 μg ml^−1^) was used to stain cells on the bottom side, and they were counted using a fluorescent microscope. For neutrophils, cells that migrated into the lower chamber were collected and counted using a haemocytometer. The data are presented as the fold change, calculated by dividing the number of cells that migrated in response to treatments by the number of cells that migrated spontaneously (migration media only).

### Efferocytosis

An efferocytosis assay kit (Cayman, 601770) was used following the manufacturer’s instructions. Neutrophils were labelled with CFSE and cultured in RPMI with 2% serum for 12 h to induce cell death. Bone marrow-derived macrophages cultured for 7 days were seeded at a density of 4 × 10^5^ cells per well in a 6-well plate with DMEM/F12 containing 10% FBS and 100 units ml^−1^ penicillin/streptomycin. Prior to the assay, macrophages were pre-treated with CGRP (1 or 20 nM) for 24 h. Macrophages were collected, labelled with CytoTell Blue, and then incubated with CFSE-labelled dead/dying neutrophils at different ratios (1:1, 1:2, and 1:4) at 37 °C for 15 min. The reaction was stopped by washing cells with ice-cold PBS containing 5% FBS and 1 mM EDTA. Cells were analysed with BD LSR Fortessa X-20 and FlowJo software (BD Biosciences). Macrophages were identified by CytoTell Blue-positive staining, and the efferocytosis index was calculated as the percentage of CFSE-positive cells in CytoTell Blue-labelled macrophages.

### Adoptive transfer of tdTomato^+^ cells for in vivo migration and efferocytosis

tdTomato^+^ bone marrow cells from *CMV-cre/Rosa26*^*tdTomato*^ male mice (8- to 12-week-old) were adoptively transferred into *Nav1.8*^*cre*^*/Rosa26*^*DTA*^ and *Rosa26*^*DTA*^ mice either directly after red blood cell lysis (migration assay) or following neutrophil isolation (efferocytosis assay). In the migration assay, 1 × 10^7^ cells were intravenously delivered on day 2 after skin or muscle injury. On day 3, collected tissues were analysed via flow cytometry to detect tdTomato^+^ cells. For the efferocytosis assay, neutrophils were cultured in low serum (2%) for 24 h to induce cell death, and 2 × 10^6^ dead or dying neutrophils were intradermally injected at the skin wound border on day 3 post-injury. After 30 min, collected tissues were assessed via flow cytometry to quantify efferocytosis as the number of monocytes or macrophages that had taken up tdTomato^+^ apoptotic neutrophils. Results were presented as fold change relative to *Rosa26*^*DTA*^ control mice.

### RT–PCR, qPCR and RNA-seq

Isolated neutrophils were treated with CGRP (1 nM) in RPMI with 10% FBS and 100 units ml^−1^ penicillin/streptomycin for 4 h at 37 °C with 5% CO_2_. Isolated monocytes cultured in DMEM/F12 with 10% FBS, 100 units ml^−1^ penicillin/streptomycin, and M-CSF (10 ng ml^−1^) for 3 days, had their medium replaced with CGRP (1 nM) for 4 h at 37 °C with 5% CO_2_. After collection, RNA extraction used the RNeasy Plus Micro Kit (Qiagen). For PCR with reverse transcription (RT–PCR) and quantitative PCR (qPCR), reverse transcription used ReverTra Ace (Toyobo). RT–PCR primers were: Human_Calcrl 5′-CATGCACATCCTTATGCAC-3′ and 5′-CCATCACTGATTGTTGACAC-3′; Human_Ramp1 5′-GCCAGGAGGCTAACTACG-3′ and 5′-GAAGAACCTGTCCACCTCTG-3′; Mouse_Calcrl 5′-GGTACCACTACTTGGCATTG-3′ and 5′-GTCACTGATTGTTGACACTG-3′; Mouse_Ramp1 5′-GACGCTATGGTGTGACT-3′ and 5′-GAGTGCAGTCATGAGCAG-3′. Human or mouse *GAPDH* primers were from Integrated DNA Technologies (51-01-07-12 and 51-01-07-13, respectively). PCR products were analysed by gel electrophoresis. qPCR was performed using LightCycle96 with software LightCycle96 (Roche Diagnostics) and TaqMan Assay primers from Thermo Fisher Scientific (*Thbs1*, Mm00449032_g1; *Gapdh*, Mm99999915_g1). For RNA-seq, RNA quantity and quality assessment, library preparation and sequencing were performed at the Medical Genomics Facility, Monash Health Translation Precinct (MHTP). RNA quantity was assessed using Qubit. RNA samples (20 ng) with RNA integrity number (RIN) value ≥ 7 were used for library preparation. First strand synthesis was performed using a dT primer which adds the Illumina P7 (5′-CAAGCAGAAGACGGCATACGAGAT-3′), 8-bp i7 index for each sample and a 10-bp unique molecular identifier. The modified reverse transcriptase reaction also adds a template switching sequence at the 5′ end of the RNA during the generation of indexed cDNA. These first stand indexed cDNA were pooled and amplified using primers to P7 and the template switch sequence. Illumina P5 was added by tagmentation by Nextera transposase during amplification. Standard Illumina R1 primer was used (main cDNA read), followed by standard i7 primer for index or unique molecular identifier. R2 primer was present but not used as it will read into poly-A tail. Sequencing was performed on the NextSeq2000 (Illumina), using NextSeq 1000/2000 P2 Reagents (100 cycles) v3 (Illumina) in accordance with the Illumina Protocol 1000000109376 v3 Nov2020.

### Demultiplexing and mapping

Fastq files were processed using the nfCore/RNAseq (v3.2) pipeline using the umi function^62^. Reads were aligned to the *Mus musculus* GRCm38 reference using STAR aligner^63^. Reads were quantified using featureCounts producing the raw genes count matrix and various quality control metrics which were summarized in a multiQC report^64,65^. Raw counts were analysed with Degust^66^, a web tool which performs normalization using trimmed mean of M values (TMM)^67^. Differential gene expression analysis was performed using limma/voom^68^ in Degust and genes with a FDR-adjusted *P* value < 0.05 were considered significantly upregulated or downregulated. Volcano plots were made using the web tool, VolcaNoseR^69^. Gene ontology enrichment analysis for biological processes was performed with the web tool, ShinyGO 0.77, by providing all upregulated or downregulated DEGs separately as the input for each experimental group^70^.

### siRNA-mediated knockdown

Macrophages (4 × 10^5^ cells per well in a 6-well plate) were transfected with 10 nM scrambled siRNA (Silencer Select Negative Control No. 1 siRNA, Thermo Fisher, 4390843) or Silencer Select Pre-Designed siRNA against mouse TSP-1 (Thermo Fisher, s124596) using Reduced-Serum Medium (Opti-MEM, Gibco) and Lipofectamine RNAiMAX (Invitrogen, 51985034) for 6 h. The medium was then replaced with fresh culture medium (DMEM/F12 with 10% FBS). After 24 h, cells were collected for the migration assay. For the efferocytosis assay, cells were cultured with 1 nM CGRP immediately after transfection. After 24 h, cells were collected and co-cultured with dead or dying neutrophils. The evaluation of cell death and polarization used the same methods as those for assessing macrophage death and polarization marker expression.

### CGRP variants

CGRP and eCGRP were synthesized by ProteoGenix. eCGRP was designed to contain PlGF residues 123–141 at the N terminus followed by a plasmin-sensitive sequence from vitronectin (KGYR)^71^. For both variants, a disulfide bond was formed between the two cysteine residues and the C-terminal phenylalanine was amidated. Peptide purity, determined by high performance liquid chromatography, was 89.63% for CGRP and 87.33% for eCGRP.

### Cleavage of eCGRP by plasmin

CGRP (4 μg) and equimolar eCGRP in 20 μl of PBS (pH 7.2) were incubated with plasmin (0.0005 U μg^−1^, Sigma) at 37 °C for 60 min. Aprotinin (25 μg ml^−1^, Sigma) was added for 5 min at 37 °C to stop plasmin activity. Samples were analysed by SDS–PAGE.

### Retention of CGRP and eCGRP into skin and muscle

CGRP (1 μg) or an equal molar amount of eCGRP was intradermally administered to the shaved dorsal skin of male 10- to 12-week-old *Nav1.8*^*cre*^^*+/−*^*/Rosa26*^*tdT+/−*^ mice, with injection sites marked using a marker. For muscle, CGRP variants were injected into the quadriceps. After 24 h, collected injection sites underwent cryosectioning and immunostaining. Fiji^59^ was used for analysis, excluding the co-localization area of CGRP with tdTomato fluorescence, indicating endogenous CGRP expression.

### cAMP quantification

Freshly isolated neutrophils or bone marrow-derived macrophages (1 million cells) were treated with CGRP (1 nM) in RPMI with 10% FBS for 30 min at 37 °C with 5% CO_2_. cAMP levels were quantified using a cAMP ELISA kit from Cayman Chemical (581001) according to the manufacturer’s instructions.

### Spontaneous pain behaviour assessment

Eight mice per group (4 males, 4 females, C57BL/6 J, 10- to 12-week-old) were acclimatized for 1 h in empty cages. The right hind paw received an intraplantar injection 1 μg of wild-type CGRP, equimolar amount of eCGRP, 0.05% capsaicin (Sigma, M2028), or 20 μl saline. Mice were immediately placed in the cage, and their behaviour was recorded. The number of episodes and the time spent licking, shaking, flinching and lifting the paw were recorded for first 5 min and for 5 min after 1, 6, 24 and 48 h.

### Hot plate test

Eight mice per group (4 males, 4 females, C57BL/6 J, 10- to 12-week-old) received an intraplantar injection of 1 μg of wild-type CGRP, equimolar amount of eCGRP, or 20 μl saline in the right hind paw. After 30 min, mice were individually placed on a metal hot plate set to 52 °C. The latency, from mouse placement on the surface to the first behavioural sign of nociception (for example, lifting, shaking, licking the hind paw or jumping), was measured. Mice were immediately removed from the hot plate after responding or after a 30 s cut-off. The test was repeated after 1, 6, 24 and 48 h.

### ELISAs for cytokines and MMPs

Homogenized skin wound and muscle tissues were incubated for 30 min on ice in T-PER Tissue Protein Extraction Reagent (10 ml per g of tissue, Thermo Fisher Scientific) containing 1 tablet of protease inhibitor for 7 ml (Roche). Samples were then centrifuged at 10,000*g* for 5 min and supernatants were stored at −80 °C. Total protein concentration was measured with a Bradford assay (Millipore). Cytokines and MMPs were detected by ELISA from R&D Systems; Mouse IL-1 beta/IL-1F2 DuoSet ELISA; Mouse CCL2/JE/MCP-1 DuoSet ELISA, Mouse CXCL2/MIP-2 DuoSet ELISA; Total MMP-2 Quantikine ELISA Kit; Mouse Total MMP-9 DuoSet ELISA.

### Statistical analysis

Statistical analyses were performed using GraphPad Prism 10 (GraphPad). Significant differences were calculated with Student’s *t*-test, one-sample *t*-test, and by ANOVA when performing multiple comparisons between groups. *P* < 0.05 was considered as a statistically significant difference.

### Reporting summary

Further information on research design is available in the Nature Portfolio Reporting Summary linked to this article.

## Online content

Any methods, additional references, Nature Portfolio reporting summaries, source data, extended data, supplementary information, acknowledgements, peer review information; details of author contributions and competing interests; and statements of data and code availability are available at 10.1038/s41586-024-07237-y.

## Supplementary information

Supplementary Figure 1 Original gel images for Extended Data Figs. 3f, 5a and 7b.

Reporting Summary

## Source data

Source Data Fig. 1

Source Data Fig. 2

Source Data Fig. 3

Source Data Fig. 4

Source Data Extended Data Fig. 1

Source Data Extended Data Fig. 2

Source Data Extended Data Fig. 3

Source Data Extended Data Fig. 4

Source Data Extended Data Fig. 5

Source Data Extended Data Fig. 6

Source Data Extended Data Fig. 7

Source Data Extended Data Fig. 8

Source Data Extended Data Fig. 9

## Data Availability

All data supporting the findings of this study are provided within the manuscript and its Supplementary Information. RNA-seq data generated for this study are deposited in the NCBI Gene Expression Omnibus under accession GSE255049. Source data are provided with this paper.

## References

[1] Julier, Z., Park, A. J., Briquez, P. S. & Martino, M. M. Promoting tissue regeneration by modulating the immune system. *Acta Biomater.* **53**, 13–28 (2017). 28119112 10.1016/j.actbio.2017.01.056

[2] Larouche, J., Sheoran, S., Maruyama, K. & Martino, M. M. Immune regulation of skin wound healing: mechanisms and novel therapeutic targets. *Adv. Wound Care* **7**, 209–231 (2018). 10.1089/wound.2017.0761 PMC6032665 29984112

[3] Muire, P. J., Mangum, L. H. & Wenke, J. C. Time course of immune response and immunomodulation during normal and delayed healing of musculoskeletal wounds. *Front. Immunol.* **11**, 1056 (2020). 32582170 10.3389/fimmu.2020.01056 PMC7287024

[4] Hanc, P., Messou, M. A., Wang, Y. & von Andrian, U. H. Control of myeloid cell functions by nociceptors. *Front. Immunol.* **14**, 1127571 (2023). 37006298 10.3389/fimmu.2023.1127571 PMC10064072

[5] Chiu, I. M. et al. Bacteria activate sensory neurons that modulate pain and inflammation. *Nature* **501**, 52–57 (2013). 23965627 10.1038/nature12479 PMC3773968

[6] Riol-Blanco, L. et al. Nociceptive sensory neurons drive interleukin-23-mediated psoriasiform skin inflammation. *Nature* **510**, 157–161 (2014). 24759321 10.1038/nature13199 PMC4127885

[7] Maruyama, K. et al. Nociceptors boost the resolution of fungal osteoinflammation via the TRP channel–CGRP–Jdp2 axis. *Cell Rep.* **19**, 2730–2742 (2017). 28658621 10.1016/j.celrep.2017.06.002

[8] Pinho-Ribeiro, F. A. et al. Blocking neuronal signaling to immune cells treats streptococcal invasive infection. *Cell* **173**, 1083–1097.e1022 (2018). 29754819 10.1016/j.cell.2018.04.006 PMC5959783

[9] Baral, P. et al. Nociceptor sensory neurons suppress neutrophil and gammadelta T cell responses in bacterial lung infections and lethal pneumonia. *Nat. Med.* **24**, 417–426 (2018). 29505031 10.1038/nm.4501 PMC6263165

[10] Yang, D. et al. Nociceptor neurons direct goblet cells via a CGRP–RAMP1 axis to drive mucus production and gut barrier protection. *Cell* **185**, 4190–4205.e4125 (2022). 36243004 10.1016/j.cell.2022.09.024 PMC9617795

[11] Hanc, P. et al. Multimodal control of dendritic cell functions by nociceptors. *Science* **379**, eabm5658 (2023). 36996219 10.1126/science.abm5658 PMC10642951

[12] Pinho-Ribeiro, F. A. et al. Bacteria hijack a meningeal neuroimmune axis to facilitate brain invasion. *Nature* **615**, 472–481 (2023). 36859544 10.1038/s41586-023-05753-x PMC10593113

[13] Kumar, A. & Brockes, J. P. Nerve dependence in tissue, organ, and appendage regeneration. *Trends Neurosci.* **35**, 691–699 (2012). 22989534 10.1016/j.tins.2012.08.003

[14] Buckley, G., Wong, J., Metcalfe, A. D. & Ferguson, M. W. Denervation affects regenerative responses in MRL/MpJ and repair in C57BL/6 ear wounds. *J Anat* **220**, 3–12 (2012). 22066944 10.1111/j.1469-7580.2011.01452.x PMC3248659

[15] Simoes, M. G. et al. Denervation impairs regeneration of amputated zebrafish fins. *BMC Dev. Biol.* **14**, 49 (2014). 25551555 10.1186/s12861-014-0049-2 PMC4333893

[16] Rinkevich, Y. et al. Clonal analysis reveals nerve-dependent and independent roles on mammalian hind limb tissue maintenance and regeneration. *Proc. Natl Acad. Sci. USA* **111**, 9846–9851 (2014). 24958860 10.1073/pnas.1410097111 PMC4103362

[17] Rabiller, L. et al. Pain sensing neurons promote tissue regeneration in adult mice. *NPJ Regen. Med.* **6**, 63 (2021). 34650070 10.1038/s41536-021-00175-7 PMC8516997

[18] Hoeffel, G. et al. Sensory neuron-derived TAFA4 promotes macrophage tissue repair functions. *Nature* **594**, 94–99 (2021). 34012116 10.1038/s41586-021-03563-7

[19] Dubin, A. E. & Patapoutian, A. Nociceptors: the sensors of the pain pathway. *J. Clin. Invest.* **120**, 3760–3772 (2010). 21041958 10.1172/JCI42843 PMC2964977

[20] Talbot, S., Foster, S. L. & Woolf, C. J. Neuroimmunity: physiology and pathology. *Annu. Rev. Immunol.* **34**, 421–447 (2016). 26907213 10.1146/annurev-immunol-041015-055340

[21] Baral, P., Udit, S. & Chiu, I. M. Pain and immunity: implications for host defence. *Nat. Rev. Immunol.* **19**, 433–447 (2019). 30874629 10.1038/s41577-019-0147-2 PMC6700742

[22] Chazaud, B. Inflammation and skeletal muscle regeneration: leave it to the macrophages! *Trends Immunol.* **41**, 481–492 (2020). 32362490 10.1016/j.it.2020.04.006

[23] Ratnayake, D. et al. Macrophages provide a transient muscle stem cell niche via NAMPT secretion. *Nature* **591**, 281–287 (2021). 33568815 10.1038/s41586-021-03199-7

[24] Abrahamsen, B. et al. The cell and molecular basis of mechanical, cold, and inflammatory pain. *Science* **321**, 702–705 (2008). 18669863 10.1126/science.1156916

[25] Martino, M. M. et al. Growth factors engineered for super-affinity to the extracellular matrix enhance tissue healing. *Science* **343**, 885–888 (2014). 24558160 10.1126/science.1247663

[26] Tan, J. L. et al. Restoration of the healing microenvironment in diabetic wounds with matrix-binding IL-1 receptor antagonist. *Commun. Biol.* **4**, 422 (2021). 33772102 10.1038/s42003-021-01913-9 PMC7998035

[27] Donnelly, C. R., Chen, O. & Ji, R. R. How do sensory neurons sense danger signals? *Trends Neurosci.* **43**, 822–838 (2020). 32839001 10.1016/j.tins.2020.07.008 PMC7530006

[28] Udit, S., Blake, K. & Chiu, I. M. Somatosensory and autonomic neuronal regulation of the immune response. *Nat. Rev. Neurosci.* **23**, 157–171 (2022). 34997214 10.1038/s41583-021-00555-4 PMC9539447

[29] Tsujikawa, K. et al. Hypertension and dysregulated proinflammatory cytokine production in receptor activity-modifying protein 1-deficient mice. *Proc. Natl Acad. Sci. USA* **104**, 16702–16707 (2007). 17923674 10.1073/pnas.0705974104 PMC2034234

[30] Clausen, B. E., Burkhardt, C., Reith, W., Renkawitz, R. & Forster, I. Conditional gene targeting in macrophages and granulocytes using *LysM*^*cre*^ mice. *Transgenic Res.* **8**, 265–277 (1999). 10621974 10.1023/a:1008942828960

[31] Peiseler, M. & Kubes, P. More friend than foe: the emerging role of neutrophils in tissue repair. *J. Clin. Invest.* **129**, 2629–2639 (2019). 31205028 10.1172/JCI124616 PMC6597202

[32] Krzyszczyk, P., Schloss, R., Palmer, A. & Berthiaume, F. The role of macrophages in acute and chronic wound healing and interventions to promote pro-wound healing phenotypes. *Front. Physiol.* **9**, 419 (2018). 29765329 10.3389/fphys.2018.00419 PMC5938667

[33] Maschalidi, S. et al. Targeting SLC7A11 improves efferocytosis by dendritic cells and wound healing in diabetes. *Nature* **606**, 776–784 (2022). 35614212 10.1038/s41586-022-04754-6

[34] Rodrigues, M., Kosaric, N., Bonham, C. A., Gurtner, G. C. & Wound, Healing: a cellular perspective. *Physiol. Rev.* **99**, 665–706 (2019). 30475656 10.1152/physrev.00067.2017 PMC6442927

[35] Wynn, T. A. & Vannella, K. M. Macrophages in tissue repair, regeneration, and fibrosis. *Immunity* **44**, 450–462 (2016). 26982353 10.1016/j.immuni.2016.02.015 PMC4794754

[36] Kourtzelis, I., Hajishengallis, G. & Chavakis, T. Phagocytosis of apoptotic cells in resolution of inflammation. *Front. Immunol.* **11**, 553 (2020). 32296442 10.3389/fimmu.2020.00553 PMC7137555

[37] Cunin, P. et al. Clusterin facilitates apoptotic cell clearance and prevents apoptotic cell-induced autoimmune responses. *Cell Death Dis.* **7**, e2215 (2016). 27148688 10.1038/cddis.2016.113 PMC4917652

[38] Lopez-Dee, Z., Pidcock, K. & Gutierrez, L. S. Thrombospondin-1: multiple paths to inflammation. *Mediators Inflamm.* **2011**, 296069 (2011). 21765615 10.1155/2011/296069 PMC3134184

[39] Soto-Pantoja, D. R. et al. Thrombospondin-1 and CD47 signaling regulate healing of thermal injury in mice. *Matrix Biol.* **37**, 25–34 (2014). 24840925 10.1016/j.matbio.2014.05.003 PMC4955854

[40] Agah, A., Kyriakides, T. R., Lawler, J. & Bornstein, P. The lack of thrombospondin-1 (TSP1) dictates the course of wound healing in double-TSP1/TSP2-null mice. *Am. J. Pathol.* **161**, 831–839 (2002). 12213711 10.1016/S0002-9440(10)64243-5 PMC1867266

[41] Kaur, S. & Roberts, D. D. Emerging functions of thrombospondin-1 in immunity. *Semin. Cell Dev. Biol.* **155**, 22–31 (2023). 37258315 10.1016/j.semcdb.2023.05.008 PMC10684827

[42] Toda, M. et al. Roles of calcitonin gene-related peptide in facilitation of wound healing and angiogenesis. *Biomed. Pharmacother.* **62**, 352–359 (2008). 18430544 10.1016/j.biopha.2008.02.003

[43] Zidan, A. A. et al. Topical application of calcitonin gene-related peptide as a regenerative, antifibrotic, and immunomodulatory therapy for corneal injury. *Commun. Biol.* **7**, 264 (2024). 38438549 10.1038/s42003-024-05934-y PMC10912681

[44] Legrand, J. M. D. & Martino, M. M. Growth factor and cytokine delivery systems for wound healing. *Cold Spring Harb. Perspect. Biol.* **14**, a041234 (2022). 35667794 10.1101/cshperspect.a041234 PMC9341469

[45] Julier, Z. et al. Enhancing the regenerative effectiveness of growth factors by local inhibition of interleukin-1 receptor signaling. *Sci. Adv.* **6**, eaba7602 (2020). 32582857 10.1126/sciadv.aba7602 PMC7292637

[46] Shi, X. et al. Neuropeptides contribute to peripheral nociceptive sensitization by regulating interleukin-1β production in keratinocytes. *Anesth. Analg.* **113**, 175–183 (2011). 21596883 10.1213/ANE.0b013e31821a0258 PMC3123433

[47] Levy, D. M. et al. Immunohistochemical measurements of nerves and neuropeptides in diabetic skin: relationship to tests of neurological function. *Diabetologia* **35**, 889–897 (1992). 1397786 10.1007/BF00399938

[48] Pittenger, G. L. et al. Intraepidermal nerve fibers are indicators of small-fiber neuropathy in both diabetic and nondiabetic patients. *Diabetes Care* **27**, 1974–1979 (2004). 15277426 10.2337/diacare.27.8.1974

[49] Pradhan, L., Nabzdyk, C., Andersen, N. D., LoGerfo, F. W. & Veves, A. Inflammation and neuropeptides: the connection in diabetic wound healing. *Expert Rev. Mol. Med.* **11**, e2 (2009). 19138453 10.1017/S1462399409000945 PMC3708299

[50] Volmer-Thole, M. & Lobmann, R. Neuropathy and diabetic foot syndrome. *Int. J. Mol. Sci.* **17**, 917 (2016). 27294922 10.3390/ijms17060917 PMC4926450

[51] Parasoglou, P., Rao, S. & Slade, J. M. Declining skeletal muscle function in diabetic peripheral neuropathy. *Clin. Ther.* **39**, 1085–1103 (2017). 28571613 10.1016/j.clinthera.2017.05.001 PMC5503477

[52] Sullivan, K. A. et al. Mouse models of diabetic neuropathy. *Neurobiol. Dis.* **28**, 276–285 (2007). 17804249 10.1016/j.nbd.2007.07.022 PMC3730836

[53] Nguyen, M. H., Cheng, M. & Koh, T. J. Impaired muscle regeneration in ob/ob and db/db mice. *ScientificWorldJournal* **11**, 1525–1535 (2011). 21805021 10.1100/tsw.2011.137 PMC5720064

[54] Wilgus, T. A., Roy, S. & McDaniel, J. C. Neutrophils and wound repair: positive actions and negative reactions. *Adv. Wound Care* **2**, 379–388 (2013). 10.1089/wound.2012.0383 PMC3763227 24527354

[55] Wong, S. L. et al. Diabetes primes neutrophils to undergo NETosis, which impairs wound healing. *Nat. Med.* **21**, 815–819 (2015). 26076037 10.1038/nm.3887 PMC4631120

[56] Chen, J. et al. Targeting matrix metalloproteases in diabetic wound healing. *Front. Immunol.* **14**, 1089001 (2023). 36875064 10.3389/fimmu.2023.1089001 PMC9981633

[57] Wetzler, C., Kampfer, H., Stallmeyer, B., Pfeilschifter, J. & Frank, S. Large and sustained induction of chemokines during impaired wound healing in the genetically diabetic mouse: prolonged persistence of neutrophils and macrophages during the late phase of repair. *J. Invest. Dermatol.* **115**, 245–253 (2000). 10951242 10.1046/j.1523-1747.2000.00029.x

[58] Jusek, G., Reim, D., Tsujikawa, K. & Holzmann, B. Deficiency of the CGRP receptor component RAMP1 attenuates immunosuppression during the early phase of septic peritonitis. *Immunobiology* **217**, 761–767 (2012). 22656887 10.1016/j.imbio.2012.04.009

[59] Schindelin, J. et al. Fiji: an open-source platform for biological-image analysis. *Nat. Methods* **9**, 676–682 (2012). 22743772 10.1038/nmeth.2019 PMC3855844

[60] Adler, J. & Parmryd, I. Colocalization analysis in fluorescence microscopy. *Methods Mol. Biol.* **931**, 97–109 (2013). 23026999 10.1007/978-1-62703-056-4_5

[61] Price, T. J. & Flores, C. M. Critical evaluation of the colocalization between calcitonin gene-related peptide, substance P, transient receptor potential vanilloid subfamily type 1 immunoreactivities, and isolectin B4 binding in primary afferent neurons of the rat and mouse. *J. Pain* **8**, 263–272 (2007). 17113352 10.1016/j.jpain.2006.09.005 PMC1899162

[62] Patel, H., Ewels, P. & Peltzer, A. nf-core/rnaseq: nf-core/rnaseq v3.2 - Copper Flamingo. *Zenodo*10.5281/zenodo.1400710 (2021).

[63] Dobin, A. et al. STAR: ultrafast universal RNA-seq aligner. *Bioinformatics* **29**, 15–21 (2013). 23104886 10.1093/bioinformatics/bts635 PMC3530905

[64] Liao, Y., Smyth, G. K. & Shi, W. featureCounts: an efficient general purpose program for assigning sequence reads to genomic features. *Bioinformatics* **30**, 923–930 (2014). 24227677 10.1093/bioinformatics/btt656

[65] Ewels, P., Magnusson, M., Lundin, S. & Kaller, M. MultiQC: summarize analysis results for multiple tools and samples in a single report. *Bioinformatics* **32**, 3047–3048 (2016). 27312411 10.1093/bioinformatics/btw354 PMC5039924

[66] Powell, D. R. drpowell/degust 4.1.1. *Zenodo*10.5281/zenodo.3258932 (2019).

[67] Robinson, M. D. & Oshlack, A. A scaling normalization method for differential expression analysis of RNA-seq data. *Genome Biol.* **11**, R25 (2010). 20196867 10.1186/gb-2010-11-3-r25 PMC2864565

[68] Law, C. W., Chen, Y., Shi, W. & Smyth, G. K. voom: precision weights unlock linear model analysis tools for RNA-seq read counts. *Genome Biol.* **15**, R29 (2014). 24485249 10.1186/gb-2014-15-2-r29 PMC4053721

[69] Goedhart, J. & Luijsterburg, M. S. VolcaNoseR is a web app for creating, exploring, labeling and sharing volcano plots. *Sci. Rep.* **10**, 20560 (2020). 33239692 10.1038/s41598-020-76603-3 PMC7689420

[70] Ge, S. X., Jung, D. & Yao, R. ShinyGO: a graphical gene-set enrichment tool for animals and plants. *Bioinformatics* **36**, 2628–2629 (2020). 31882993 10.1093/bioinformatics/btz931 PMC7178415

[71] Chain, D., Kreizman, T., Shapira, H. & Shaltiel, S. Plasmin cleavage of vitronectin. Identification of the site and consequent attenuation in binding plasminogen activator inhibitor-1. *FEBS Lett.* **285**, 251–256 (1991). 1713175 10.1016/0014-5793(91)80810-p

