# Supplementary material for: CGRP sensory neurons promote tissue healing via neutrophils and macrophages
Source: Nature. 2024 Mar 27;628(8008):604–11. doi: 10.1038/s41586-024-07237-y (PMC11023938; doi:10.1038/s41586-024-07237-y)
Supplement: Supplementary file 1 — Original gel images for Extended Data Figs. 3f, 5a and 7b. [file 41586_2024_7237_MOESM1_ESM.pdf]

nature portfolio

https://doi.org/10.1038/s41586-024-07237-y

Supplementary information

CGRP sensory neurons promote tissue healing via neutrophils and macrophages

In the format provided by the authors and unedited

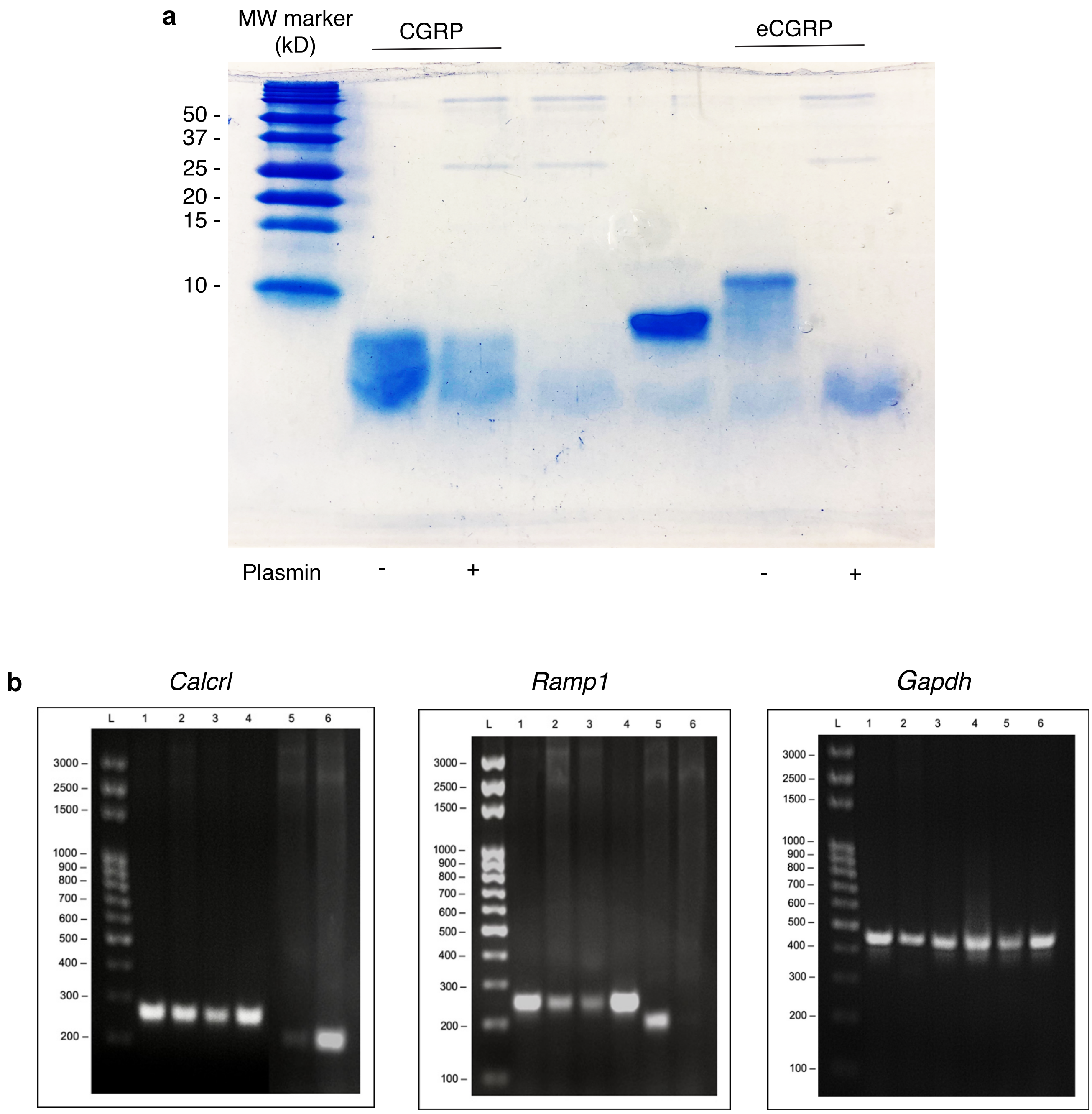

L: Ladder
Lane 1: Macrophages
Lane 2: Fibroblasts
Lane 3: Myoblasts
Lane 4: Neutrophils
Lane 5: Keratinocytes
Lane 6: Endothelial cells
