# Supplementary material for: CGRP sensory neurons promote tissue healing via neutrophils and macrophages
Source: Nature. 2024 Mar 27;628(8008):604–11. doi: 10.1038/s41586-024-07237-y (PMC11023938; doi:10.1038/s41586-024-07237-y)
Supplement: Supplementary file 2 — Reporting Summary [file 41586_2024_7237_MOESM2_ESM.pdf]

nature portfolio

Corresponding author(s): Mikaël M. Martino

Last updated by author(s): Feb 19, 2024

Reporting Summary

Nature Portfolio wishes to improve the reproducibility of the work that we publish. This form provides structure for consistency and transparency in reporting. For further information on Nature Portfolio policies, see our Editorial Policies and the Editorial Policy Checklist.

Statistics

For all statistical analyses, confirm that the following items are present in the figure legend, table legend, main text, or Methods section.

n/a	Confirmed	
☐	☒	The exact sample size (*n*) for each experimental group/condition, given as a discrete number and unit of measurement
☐	☒	A statement on whether measurements were taken from distinct samples or whether the same sample was measured repeatedly
☐	☒	The statistical test(s) used AND whether they are one- or two-sided<br>*Only common tests should be described solely by name; describe more complex techniques in the Methods section.*
☐	☒	A description of all covariates tested
☐	☒	A description of any assumptions or corrections, such as tests of normality and adjustment for multiple comparisons
☐	☒	A full description of the statistical parameters including central tendency (e.g. means) or other basic estimates (e.g. regression coefficient) AND variation (e.g. standard deviation) or associated estimates of uncertainty (e.g. confidence intervals)
☐	☒	For null hypothesis testing, the test statistic (e.g. *F*, *t*, *r*) with confidence intervals, effect sizes, degrees of freedom and *P* value noted<br>*Give P values as exact values whenever suitable.*
☒	☐	For Bayesian analysis, information on the choice of priors and Markov chain Monte Carlo settings
☒	☐	For hierarchical and complex designs, identification of the appropriate level for tests and full reporting of outcomes
☒	☐	Estimates of effect sizes (e.g. Cohen's *d*, Pearson's *r*), indicating how they were calculated

Our web collection on statistics for biologists contains articles on many of the points above.

Software and code

Policy information about availability of computer code

Data collection	Leica AF6000LX and DMi8inverted Fluorescence microscope and Leica SP8 inverted confocal microscope for scanning immunostained slides.<br>BD Fortessa for flow cytometery analysis.<br>NextSeq2000 (Illumina) for RNA sequencing.<br>PerkinElmer EnVision multi mode plate reader with EnSpire Manager software v2.00 for proliferation assay.<br>LightCycle96 with software LightCycle96 SW 1.1 (Roche Diagnostics).<br>C1000 Touch Thermal Cycler for complementary DNA (cDNA) preparation.
Data analysis	Statistics: GraphPad Prism software (v9.3.1).<br>Histology and immunofluorescence analysis: Fiji open source software (v2.14.0/1.54f) and Aperio ImageScope Viewer (v12.4.6.5003).<br>Flow cytometery analysis: FlowJo v10.<br>RNA sequencing analysis: STAR aligner (v2.7.0a), VolcaNoseR web tool (v1.0.3), Degust web tool (v41.1), and ShinyGO (v0.77) web tool.

For manuscripts utilizing custom algorithms or software that are central to the research but not yet described in published literature, software must be made available to editors and reviewers. We strongly encourage code deposition in a community repository (e.g. GitHub). See the Nature Portfolio guidelines for submitting code & software for further information.

Data

Policy information about availability of data

All manuscripts must include a data availability statement. This statement should provide the following information, where applicable:

- Accession codes, unique identifiers, or web links for publicly available datasets
- A description of any restrictions on data availability
- For clinical datasets or third party data, please ensure that the statement adheres to our policy

All data supporting the findings of this study are found within the manuscript and its Supplementary Information. Source data are provided with this paper. RNA sequencing data generated for this study are deposited in NCBI's Gene Expression Omnibus database (GSE255049). RNA-seq reads were were mapped to the Mus musculus GRCm38.p6 genome.

Research involving human participants, their data, or biological material

Policy information about studies with human participants or human data. See also policy information about sex, gender (identity/presentation), and sexual orientation and race, ethnicity and racism.

Reporting on sex and gender	N/A
Reporting on race, ethnicity, or other socially relevant groupings	N/A
Population characteristics	N/A
Recruitment	N/A
Ethics oversight	N/A

Note that full information on the approval of the study protocol must also be provided in the manuscript.

Field-specific reporting

Please select the one below that is the best fit for your research. If you are not sure, read the appropriate sections before making your selection.

☒ Life sciences ☐ Behavioural & social sciences ☐ Ecological, evolutionary & environmental sciences

For a reference copy of the document with all sections, see nature.com/documents/nr-reporting-summary-flat.pdf

Life sciences study design

All studies must disclose on these points even when the disclosure is negative.

Sample size	No statistical methods were used to predetermine sample size. Estimates were made based on our previous experience, availability and feasibility required to obtain statistically significant results: Martino, M. M. et al. Growth factors engineered for super-affinity to the extracellular matrix enhance tissue healing. Science 343, 885-888 (2014); Julier, Z. et al. Enhancing the regenerative effectiveness of growth factors by local inhibition of interleukin-1 receptor signaling. Sci Adv 6, eaba7602 (2020); Tan, J. L. et al. Restoration of the healing microenvironment in diabetic wounds with matrix-binding IL-1 receptor antagonist. Commun Biol 4, 422 (2021).<br>This research comply with the 3Rs rule on reducing, replacing and refining the use of animals for scientific purpose. The simple size (n) for each experiment is provided in the figure legends.
Data exclusions	Data were not excluded from study reporting. Animals that showed signs of suffering were not included in study reporting.
Replication	Results shown are representative of at least 3 independently performed experiments. Each dot in the bar plots indicates a separate animal. All attempts at replication were successful.
Randomization	For all in vivo and in vitro experiments involving cells from animals, mice were preselected based on age and then randomly assigned to treatment groups.
Blinding	The experimenters were blinded to the mouse genotype or treatment conditions during surgical procedures. Histology and immunohistological analyses were blindly performed. Blinding was not performed for all other experiments due to availability of researchers.

Reporting for specific materials, systems and methods

We require information from authors about some types of materials, experimental systems and methods used in many studies. Here, indicate whether each material, system or method listed is relevant to your study. If you are not sure if a list item applies to your research, read the appropriate section before selecting a response.

Materials & experimental systems

n/a	Involved in the study
☐	☒ Antibodies
☐	☒ Eukaryotic cell lines
☒	☐ Palaeontology and archaeology
☐	☒ Animals and other organisms
☒	☐ Clinical data
☒	☐ Dual use research of concern
☒	☐ Plants

Methods

n/a	Involved in the study
☒	☐ ChIP-seq
☐	☒ Flow cytometry
☒	☐ MRI-based neuroimaging

Antibodies

Antibodies used

Anti-mouse antibodies used in flow Cytometry in vivo:
1. FITC anti-CD11b (BioLegend, Clone M1/70, 6.6 μg/ml).
2. BV711 anti-CD11b (BioLegend, Clone M1/70, 2 μg/ml).
3. PE anti-F4/80 (BioLegend, Clone BM8, 4 μg/ml).
4. BV421 anti-Ly6G (BioLegend, Clone 1A8, 2 μg/ml).
5. BV711 anti-Ly6C (BioLegend,clone HK1.4, 1 μg/ml).
6. FITC anti-Ly6C (BioLegend,clone HK1.4, 5 μg/ml).
7. APC anti-CD206 (BioLegend, clone C068C2, 2 μg/ml).
8. PE-Cyanine7,anti-CD206 (BioLegend, clone C068C2, 2.6μg/ml).
9. BV711 anti-CD3 (BioLegend, clone 17A2, 4 μg/ml).
10. PE-Cyanine7 anti-CD3 (BioLegend, clone 17A2, 4 μg/ml).
11. APC anti-CD4 (BioLegend, clone GK1.5, 2 μg/ml).
12. BV421 anti-CD8 (BioLegend, clone 53-6.7, 2 μg/ml).
13. PE anti-TCRβ (clone H57-597, 2 μg/ml).
14. APC/Fire 750 anti-TCR γ/δ (clone GL3, 2 μg/ml).
15. PE-Cyanine7 anti-CD11c (clone N418, 2 μg/ml).
16. APC/Fire 750 anti-MHC Class II (clone M5/114.15.2, 2 μg/ml).

Anti-mouse antibodies used in flow cytometry in vitro:
1. PE anti-CD11b (BioLegend, clone M1/70, 1 μg/ml).
2. BV711 anti-F4/80 (BioLegend, cone BM8, 2 μg/ml).
3. PE-Cyanine7 anti-CD206 (BioLegend, clone C068C2, 1 μg/ml).
4. APC anti-mouse arginase 1 (ThermoFisher, Clone AlexF5, 1 μg/ml).
5. APC anti-CD80 (BioLegend, clone 16-10A1, 0.5 μg/ml).

Immunostaining antibodies:
1. Rabbit anti-CGRP (66.7 μg/ml, Sigma, #C8198).
2. Rabbit anti-substance P (1:500, Immunostar, #20064).
3. Rabbit anti-vasoactive intestinal peptide (1:500, Immunostar, #20077).
4. Goat anti-galanin (1 μg/ml, Abcam, #99452).
5. Mouse anti-thrombospodin-1 (5 μg/ml, A6.1, Thermo Fisher Scientific, #14-9756-82).
6. Rat anti-CD11b (5 μg/ml, M1/70, Thermo Fisher Scientific, #14-0112-82).
7. Unconjugated AffiniPure Fab Fragment Goat Anti-Mouse IgG (H+L) (Jackson ImmunoResearch Labs, #115-007-003, 100 μg/ml)
8. F(ab')2-Goat anti-Rabbit IgG (H+L) Cross-Adsorbed Secondary Antibody, Alexa Fluor 488 (2.6 μg/ml, Thermo Fisher Scientific, #A-11070).
9. Donkey anti-goat IgG H&L conjugated with Alexa Fluor 488 (2.6 μg/ml, Thermo Fisher Scientific, #A-11055).
10. Goat anti-mouse (IgG (H+L) Highly Cross-Adsorbed Secondary Antibody, Alexa Fluor Plus 488, 2.6 μg/ml, Thermo Fisher Scientific, #A48286TR).
11. Goat anti-Rat (IgG (H+L) Highly Cross-Adsorbed Secondary Antibody, Alexa Fluor Plus 594, 2.6 μg/ml, Thermo Fisher Scientific, #A48264).
12. Rabbit anti-Ramp-1 (8.5 μg/ml, Alomone Lab, #ARR-021).
13. Rabbit anti-calcitonin receptor like receptor (5 μg/ml, Biorbyt, #orb526584).
14. Rat anti-mouse Ki-67 (5 μg/ml, Thermo Fisher Scientific, #14-5698-82).
15. Mouse anti-mouse cytokeratin 14 (4 μg/ml, Thermo Fisher Scientific, #MA5-11599).
16. Goat anti-mouse Alexa Fluor-647 (2 μg/ml, Thermo Fisher Scientific, #A-21235).
17. Goat anti-rat Alexa Fluor-488 (2.67 μg/ml, Thermo Fisher Scientific, # A48262TR).
18. Unconjugated affinity-purified F(ab) fragment anti-mouse IgG (H+L) (10 μg/ml, Jackson ImmunoResearch, #AB_2338476).

Validation

All BioLegend primary antibodies for flow cytometry were validated by the manufacturer on C57BL/6 mouse bone marrow cells, C57BL/6 mouse splenocytes or BALB/c mouse peritoneal macrophages.
APC anti-mouse arginase 1 was validated on normal human lysed whole blood cells by the manufacturer.
Rabbit anti-CGRP was validated by the manufacturer with immunohistochemistry of trigeminal ganglia cell cultures at a dilution of 1:200 and with immunocytochemistry of mouse lung slices at a dilution of 1:500.
Rabbit anti-substance P was validated was validated by the manufacturer using soluble pre-adsorption with substance P at a final concentration of 10 μg of peptide per ml of diluted anti-substance P.

Rabbit anti-vasoactive intestinal peptide was validated by the manufacturer using soluble pre-adsorption with vasoactive intestinal peptide at a final concentration of 10-5 M.
Goat anti-galanin was validated on mouse samples by Labome (ab99452).
Mouse anti-thrombospodin-1 was validated on mouse brain tissue sections by the manufacturer.
Rat anti-CD11b (M1/70, same clone as the anti-CD11b from Biolegend) was validated by the manufacturer using immunostaining of formalin-fixed paraffin-embedded human colon adenocarcinoma tissue sections.
Rabbit anti-Ramp-1 was validated by the manufacturer using immunostaining of living rat pheochromocytoma (PC12) cells.
Rabbit anti-calcitonin receptor like receptor was validated by our lab on mouse neutrophils and macrophages.
Rat anti-mouse Ki-67 was validated by the manufacturer using immunostaining of formalin-fixed paraffin-embedded mouse thymus tissue sections.
Mouse anti-mouse cytokeratin 14 was was validated by the manufacturer using immunostaining of 70% confluent log phase A-431 cells.

Eukaryotic cell lines

Policy information about cell lines and Sex and Gender in Research

Cell line source(s)

Human umbilical vein endothelial cells were purchased from Sigma-Aldrich (Catalog #200P-05N).
C2C12 mouse myoblast cell line were purchased from Cell Bank (Australia, Code 91031101).
HaCaT keratinocyte cell line was gifted by Professor Richard Boyd (Monash University, Australia).

Authentication

HUVEC: cells were authenticated by the supplier using morphology and staining for VEGFR2 and CD31.
C2C12: cells were originally obtained from ATCC and authenticated by ATCC and our lab using morphology.
HaCat: cells were authenticated by Professor Ricard Boyd's lab and our lab using morphology.

Mycoplasma contamination

Cells obtained from vendors were certified negative for mycoplasma contamination.

Commonly misidentified lines
(See ICLAC register)

No commonly misidentified cell lines were used in the study.

Animals and other research organisms

Policy information about studies involving animals; ARRIVE guidelines recommended for reporting animal research, and Sex and Gender in Research

Laboratory animals

C57BL6/J were from from the Monash Animal Research Platform. 10-12-week-old mice were used.

Nav1.8Cre+/+ mice (B6.129-Scn10atm2(cre)Jnw/H B6, stock ID EM:04582, European Mouse Mutant Archive) were used for in vitro fertilisation to generate Nav1.8Cre+/- mice on a C57BL/6J background in house.
Rosa26DTA+/+ (B6.129-Gt(ROSA)26Sortm1(DTA)Mrc/J, strain 010527, Jackson Laboratory) were maintained on a C57BL/6J background in house.
Nav1.8Cre+/- mice were bred with Rosa26DTA+/+ to generate Nav1.8Cre+/-/Rosa26DTA+/- mice and Nav1.8Cre-/-/Rosa26DTA+/-. 10-12-week-old mice were used.

Rosa26tdT reporter mice (B6.Cg-Gt(ROSA)26Sortm14(CAG-tdTomato)Hze/J, strain 007914, Jackson Laboratory).
Rosa26tdT mice were bred with Nav1.8Cre+/+ mice to generate Nav1.8Cre+/-/Rosa26Tdt+/-. 10-12-week-old mice were used.

BKS.Cg-Dock7 m +/+ Lepr db /J (Lepr db/db) mice were obtained from the Jackson Laboratory and bred in house. 12-14-week-old mice were used.

B6.129S2-Ramp1<tm1.2Tsuj> sperm was kindly provided by Prof. Kazutake Tsujikawa (Graduate School and School of Pharmaceutical Sciences, Osaka University). B6.129S2-Ramp1<tm1.2Tsuj> sperms were used for in vitro fertilisation to generate Ramp1fl/+ mice.
Ramp1-/- mice were generated by crossing Ramp1fl/fl mice with CAGcre mice (C57BL/6-Tg(CAG-cre)13Miya, RIKEN BioResource Research Center, strain 09807). 10-12-week-old mice were used.
Ramp1fl/fl/LysMCre+/- mice were bred in house by crossing Ramp1fl/fl with LysMCre+/+ mice (B6.129P2-Lyzs<tm1(cre)Ifo>, RIKEN BioResource Research Center, strain 02302). LysMCre+/- littermates were used as controls. 10-12-week-old mice were used.

Mice constitutively expressing tdTomato were obtained by crossing with B6.C-Tg(CMV-cre)1Cgn/J (8-10-week-old, Jackson Laboratory, strain 006054) with Rosa26DTA+/+ mice.

Wild animals

No wild animals were used.

Reporting on sex

All in vitro experiments were performed by using male mouse bone marrow cells.
C57BL6/J mice: male and female.
Nav1.8Cre+/-/Rosa26DTA+/- and Nav1.8Cre-/-/Rosa26DTA+/- mice: male.
Nav1.8Cre+/-/Rosa26DTA+/- mice when treated with CGRP variants or saline: male (muscle experiments) and female (skin experiments).
Nav1.8Cre+/-/Rosa26Tdt+/- mice: male.
BKS.Cg-Dock7 m +/+ Lepr db /J (Lepr db/db)mice: male (muscle experiments) and female (skin experiments).
Ramp1-/- mice: male.
Ramp1fl/fl with LysMCre+/- mice: male.
LysMCre+/- mice: male.

Field-collected samples | No field-collected samples were used.

Ethics oversight | Animal experiments were approved by the Monash Animal Research Platform ethics committee and Animal Research Committee of the Research Institute for Microbial Diseases of Osaka University (project numbers 13294, 13335, 17075, 14013, 23006).

Note that full information on the approval of the study protocol must also be provided in the manuscript.

Flow Cytometry

Plots

Confirm that:

☒ The axis labels state the marker and fluorochrome used (e.g. CD4-FITC).

☒ The axis scales are clearly visible. Include numbers along axes only for bottom left plot of group (a 'group' is an analysis of identical markers).

☒ All plots are contour plots with outliers or pseudocolor plots.

☒ A numerical value for number of cells or percentage (with statistics) is provided.

Methodology

Sample preparation

In vivo immune cell profiling:
Skin wounds were harvested using an 8 mm biopsy punch, and muscle defects were dissected to isolate the quadriceps. Tissue samples were minced with scissors and subjected to two serial digestions with collagenase XI (1 mg/ml) at 37°C (30 minutes for skin, 20 minutes for muscle). After the first digestion, the supernatant was collected and mixed with neutralization buffer (DMEM/F12 with 5 mM EDTA). The first collection was kept on ice, and fresh collagenase XI was added to the undigested tissue for the second digestion. Digestion mixtures were passed through a 70 µm cell strainer.

In vitro macrophages:
Mouse bone marrow cells derived-macrophages were treated with different cytokines with or without CGRP (1 or 20 nM) for 24-72 hours. Cells were then detached with TrypLE (Gibco) containing 3 mM EDTA and harvested in culture medium containing 10% FBS for further analysis.

In vitro neutrophils:
Mouse neutrophils were isolated from mouse bone marrow by using EasySep Mouse Neutrophil Enrichment Kit (STEMCELL Technologies). Isolated cells were treated with different cytokines with or without CGRP (1 or 20 nM) for further analysis.

Instrument

BD Fortessa X20.

Software

FlowJo Software v10 for analysis.

Cell population abundance

There was no cell sorting experiments.

Gating strategy

All the gating strategies are based on FMOs (Fluorescence Minus One).

Skin or muscle neutrophils (CD11b+ Ly6G+) were gated from live cells.
Skin or muscle monocytes/macrophages (CD11b+F4/80+) were gated by excluding Ly6G from the live cells.
Skin or muscle Ly6Chigh cells: Ly6Chigh population was gated from CD11b+ F4/80+ cells.
CD206 expression in skin or muscle macrophages: CD206 expression was determined in the macrophage population (F4/80+ CD11b+, Ly6G-).
Skin or muscle dendritic cells (CD11c+ MHC-II+) were gated by excluding neutrophils and macrophages from the live cells.
Skin or muscle CD4 T cells (CD3+ CD4+) were gated by excluding CD11b+ from the live cells.
Skin or muscle CD8 T cells: (CD3+ CD8+) were gated by excluding CD11b+ from the live cells.
Skin or muscle γδ T cells (CD3+ TCRCγ+ TCRβ-) were gated by excluding CD11b+ from the live cells.
Skin or muscle tdTomato cells were gated using the strategy above and using FMO for tdTomato.

☒ Tick this box to confirm that a figure exemplifying the gating strategy is provided in the Supplementary Information.
